# The ORION framework for outlier-robust parameter estimation in diffusion kurtosis imaging

**DOI:** 10.1038/s41598-026-62390-w

**Published:** 2026-08-13

**Authors:** Vincenzo Anania, Ben Jeurissen, Thibo Billiet, Jan Sijbers, Arnold J. den Dekker

**Affiliations:** 1 imec - Vision Lab, Department of Physics, University of Antwerp, Antwerp, Belgium; 2 icometrix, Leuven, Belgium; 3 µNEURO Research Centre of Excellence, University of Antwerp, Antwerp, Belgium

**Keywords:** Diffusion MRI, Robust parameter estimation, Outlier detection, DKI, Least squares, Computational biology and bioinformatics, Mathematics and computing, Medical research

## Abstract

Diffusion-weighted images are susceptible to intensity outliers that degrade the accuracy and precision of diffusion tensor and diffusion kurtosis imaging (DKI) parameter estimates. In this work, we introduce ORION, a voxel-wise framework for outlier-robust DKI parameter estimation based on constrained linear least squares estimators. ORION is designed to detect intensity outliers in both diffusion-weighted and non-diffusion-weighted images, ensuring the robustness of DKI metrics against artifactual signal dropouts and signal overshoots. First, the performance of the proposed framework in terms of outlier detection and statistical parameter estimation is compared to that of the previously proposed iterative reweighted linear least squares (IRLLS) outlier-robust approach in a Monte Carlo simulation framework. Next, we compare the DKI metrics extracted from two synthetic outlier-corrupted datasets using ORION, IRLLS, and a conventional weighted linear least squares (WLLS) approach. The synthetic datasets were realistically generated from in vivo MRI data from two participants in the Alzheimer’s Disease Neuroimaging Initiative (ADNI), with one dataset affected by signal dropout and the other by signal overshoot. In comparison to WLLS, both ORION and IRLLS achieved significantly lower errors in estimating white matter DKI metrics in regions affected by intensity outliers, with ORION also outperforming IRLLS on a subset of metrics under signal dropout conditions. Finally, the DKI metrics extracted from the in vivo datasets were found to be in line with the results obtained on the synthetic data. In conclusion, ORION successfully detects and rejects intensity outliers improving upon conventional DKI estimation techniques.

## Introduction

Diffusion-weighted magnetic resonance (DW-MR) imaging is a non-invasive technique typically used to reveal the brain’s microstructural properties by probing the local diffusion of water molecules. Images are acquired by applying a diffusion-sensitizing magnetic field gradient and mathematical models are used to extract quantitative features. While the diffusion tensor imaging (DTI) model remains the most commonly applied, it has been shown that the diffusion kurtosis imaging (DKI) model can provide more accurate estimates of diffusion tensor properties^1^ as well as additional information in clinical applications^2,3^. Unfortunately, the quality of the extracted diffusion tensor and diffusion kurtosis metrics can be degraded by artifacts in the form of outliers, i.e., signal intensities that significantly deviate from the expected behavior. Such signal intensity deviations may be caused by measurement variability due to noise, subject motion, physiological mechanisms (e.g., cardiac pulsation and respiratory motion), or system instabilities, such as scanner vibration. The development of post-processing techniques that mitigate the effect of such outliers on quantitative diffusion MRI (dMRI) biomarkers has been an active area of research over the last two decades^4,5^. The main purpose of outlier correction strategies is to reduce the bias and increase the precision of dMRI metrics in areas affected by an artifactual decrease or increase of the signal.

In the last two decades, several outlier-robust diffusion parameter estimation approaches have been proposed that identify intensity outliers at a voxel level based on the distribution of the residuals of a model fit. One of the first techniques introduced for outlier-robust DTI parameter estimation is RESTORE^6^: an iterative algorithm using weighted non-linear least squares (NLLS) estimation in which, at each iteration, the influence of potential outliers is reduced through the Geman-McClure weighting function^7^. When convergence is reached, the data points whose residuals fall beyond a confidence interval of three times the expected signal standard deviation are identified as outliers and excluded from the final NLLS fit. Several other algorithms have been inspired by RESTORE. Morris et al.^8^, for example, integrated RESTORE in their correction pipeline (DROP-R) to identify outliers prior to resampling so that the averaging of outlier data points with good data can be avoided. An important difference with RESTORE is that in DROP-R the detected outliers are not removed but substituted with replacement values. Maximov et al.^9^ proposed an iterative framework in which the NLLS fitting with Geman-McClure weighting of RESTORE is replaced by a constrained non-linear least trimmed squares (LTS) fitting for conventional DTI datasets acquired with multiple gradient directions and a single diffusion weighting strength (*b*-value). Within the same framework, a constrained non-linear least median squares (LMS) fitting is proposed for datasets acquired with fewer gradient directions and multiple non-zero *b*-values. An informed version of RESTORE (i.e., iRESTORE) has been suggested based on the notion that outliers in DW images are more likely to manifest as signal dropouts than signal increases^10^.

In the last decade, two outlier-robust methods have been proposed that exploit the fact that both the DTI and the DKI model can be linearized by the natural log-transformation, which facilitates linear least squares (LLS) estimation of the model parameters. LLS approaches are often preferred over NLLS approaches due to their higher computational efficiency. Moreover, it has been shown that, if properly used, LLS diffusion parameter estimators may outperform NLLS estimators in terms of accuracy and precision^11^. The outlier-robust methods IRLLS^12^ and REKINDLE^13^, designed for the DTI and the DKI model, respectively, employ weighted linear least squares (WLLS) estimation techniques, thus providing a potentially high performance in terms of precision and accuracy^11^, including minimal bias under certain conditions^14^. For outlier detection, REKINDLE conducts linear regression after a variable transformation of the heteroscedastic diffusion signals into a homoscedastic space. On the other hand, IRLLS performs outlier detection in either the linear or the non-linear space depending on whether the residuals are negative or positive (*two-eyes* approach).

Next to voxel-wise outlier detection methods, slice-wise methods have been proposed in the literature^15–20^. Whereas voxel-wise methods are typically performed after correction for subject motion and eddy current induced geometric distortions, slice-wise approaches are applied prior to any image registration operation to avoid slice-wise intensity artifacts to spread over multiple slices^19^. Slice-wise approaches have been proposed in which identified outlier slices are completely excluded from further analysis, either individually^16,17,20^ or together with all other slices of the corresponding gradient volume^15^, as well as approaches in which identified outlier slices are replaced by Gaussian model predictions^18^, or attributed a reduced weight during model fitting^19^. In addition to purely voxel-wise or slice-wise strategies, hybrid methods have been proposed that inform voxel-wise robust fitting techniques with slice-wise information^21–23^.

In the current work, we propose a novel framework for voxel-wise outlier-robust DKI parameter estimation based on LLS estimators, which we have coined ORION (Outlier-Robust diffusIon kurtOsis imagiNg). ORION builds upon the IRLLS framework introducing key technical modifications tailored for its applicability to DKI: the removal of the goodness-of-fit (GOF) check, the targeted rejection of outliers in non-diffusion-weighted ($b_0$) images, and the use of constrained optimization to ensure physically meaningful results. Through a robust methodological design, we have validated the ORION framework across standard DKI metrics as well as white matter tract integrity (WMTI) metrics. In addition to single-voxel Monte Carlo simulations, our analysis includes realistic synthetic datasets derived from in vivo acquisitions, highlighting the prevalence of outliers in real-world dMRI measurements and the need for approaches such as ORION to ensure reliable microstructural characterization.

## Methods

### Theory

#### The DKI model

As a result of tissue heterogeneity and biological restrictions, the diffusion displacement of water molecules in most biological tissues, including the brain, has a non-Gaussian distribution. The DKI model^24^ allows us to quantify this non-Gaussian diffusion. According to the DKI model, the DW-MR signal can be expressed as:

1 $$\begin{aligned} \begin{aligned} S(b,\boldsymbol{g})&= S_{0}\text {exp} \left( -b \sum ^3_{i,j=1} g_i g_j D_{ij} + \frac{b^2}{6} \left( \sum ^3_{i=1} \frac{D_{ii}}{3} \right) ^2 \sum ^3_{i,j,k,l=1} g_i g_j g_k g_l W_{ijkl} \right) \\ &= S_{0}\text {exp} \left( -b \sum ^3_{i,j=1} g_i g_j D_{ij} + \frac{b^2}{6} \sum ^3_{i,j,k,l=1} g_i g_j g_k g_l K_{ijkl}\right) , \end{aligned} \end{aligned}$$

where $S_0$ denotes the non-diffusion-weighted signal, *b* and $\boldsymbol{g}$ denote the diffusion weighting strength and direction, respectively, $D_{ij}$ the elements of the second-order diffusion tensor, $W_{ijkl}$ the elements of the fourth-order kurtosis tensor and $K_{ijkl}=\left( \sum ^3_{i=1} \frac{D_{ii}}{3} \right) ^2 W_{ijkl} = \text {MD}^2 W_{ijkl}$—with MD the mean diffusivity—the elements of the scaled kurtosis tensor. The non-diffusion-weighted signal, the 6 independent elements of the diffusion tensor and the 15 independent elements of the scaled kurtosis tensor can be combined into the DKI model parameter vector $\boldsymbol{\theta }$ ($N_{par} \times 1$):

2 $$\begin{aligned} \begin{aligned} \boldsymbol{\theta }&= [\text {log}(S_{0}) \quad D_{xx} \quad D_{xy} \quad D_{xz} \quad D_{yy} \quad D_{yz} \quad D_{zz} \quad K_{xxxx} \quad K_{xxxy} \quad K_{xxxz} \quad K_{xxyy} \quad ... \\&K_{xxyz} \quad K_{xxzz} \quad K_{xyyy} \quad K_{xyyz} \quad K_{xyzz} \quad K_{xzzz} \quad K_{yyyy} \quad K_{yyyz} \quad K_{yyzz} \quad K_{yzzz} \quad K_{zzzz}]^{T}. \end{aligned} \end{aligned}$$

In a more compact matrix form, the DW-MR signal can thus be written as:

3 $$\begin{aligned} \boldsymbol{S}(\boldsymbol{B},\boldsymbol{\theta })=\text {exp}(\boldsymbol{B}\boldsymbol{\theta }), \end{aligned}$$

where the $\boldsymbol{B}$-matrix, which size is given by the number of diffusion-weighted images ($N_{\text {DWI}}$) *×* the number of model parameters ($N_{par}$), embodies the effect of the diffusion weighting strength and directions. For each diffusion measurement, we define the column vector $\boldsymbol{b}_{i}$ as the transpose of the *i*-th row of the $\boldsymbol{B}$-matrix:

4 $$\begin{aligned} \begin{aligned} \boldsymbol{b}_{i}&= [1 \quad -b_{i}(g_{ix}^2 \quad 2g_{ix}g_{iy} \quad 2g_{ix}g_{iz} \quad g_{iy}^2 \quad 2g_{iy}g_{iz} \quad g_{iz}^2) \quad b_{i}^2/6(g_{ix}^4 \quad 4g_{ix}^3g_{iy} \quad 4g_{ix}^3g_{iz} \quad 6g_{ix}^2g_{iy}^2 \quad ... \\&12g_{ix}^2g_{iy}g_{iz} \quad 6g_{ix}^2g_{iz}^2 \quad 4g_{ix}g_{iy}^3 \quad 12g_{ix}g_{iy}^2g_{iz} \quad 12g_{ix}g_{iy}g_{iz}^2 \quad 4g_{ix}g_{iz}^3 \quad g_{iy}^4 \quad 4g_{iy}^3g_{iz} \quad 6g_{iy}^2g_{iz}^2 \quad 4g_{iy}g_{iz}^3 \quad g_{iz}^4)]^T, \end{aligned} \end{aligned}$$

where $b_{i}$ and $\boldsymbol{g}_{i}$ define the diffusion weighting strength and direction for the *i*-th measurement, respectively. By linearizing Eq. (3) through the natural logarithm, i.e., $\text {log}(\boldsymbol{S}(\boldsymbol{B},\boldsymbol{\theta }))=\boldsymbol{B}\boldsymbol{\theta }$, the estimation of the DKI model parameters can be formulated as a linear regression problem.

#### Linear least squares estimation

Linear least squares (LLS) estimators provide an elegant and computationally efficient closed-form solution to the inverse problem of estimating the DKI model parameters, $\boldsymbol{\theta }$, from the measured noisy DW data, $\tilde{\boldsymbol{S}}(b,\boldsymbol{g})$. The ordinary LLS estimate of $\boldsymbol{\theta }$ is given by:

5 $$\begin{aligned} \hat{\boldsymbol{\theta }}_{\text {LLS}} = (\boldsymbol{B}^T\boldsymbol{B})^{-1}\boldsymbol{B}^T\boldsymbol{y}, \end{aligned}$$

where $\boldsymbol{y}$ is the linearized version of the noisy DW-MR signal, i.e., $\text {log}(\tilde{\boldsymbol{S}}(b,\boldsymbol{g}))$. However, to account for the heteroscedasticity of the log-transformed data $\boldsymbol{y}$, different weights can be introduced for each residual resulting in the weighted linear least squares (WLLS) estimator:

6 $$\begin{aligned} \hat{\boldsymbol{\theta }}_{\text {WLLS}} = (\boldsymbol{B}^T\boldsymbol{W}\boldsymbol{B})^{-1}\boldsymbol{B}^T\boldsymbol{W}\boldsymbol{y}. \end{aligned}$$

In Eq. (6), $\boldsymbol{W}$ represents the weight matrix of size $N_{\text {DWI}} \times N_{\text {DWI}}$. The linear estimator with the highest achievable accuracy is obtained when the weightings are chosen as the squares of the LLS predictions of the signal^11,25^:

7 $$\begin{aligned} \boldsymbol{W} = \text {diag}(\text {exp}(2\boldsymbol{B}\hat{\boldsymbol{\theta }}_{\text {LLS}})). \end{aligned}$$

### The outlier-robust framework

Since IRLLS serves as a baseline for comparison, in this section, we first review its key concepts and then introduce the main innovations of the ORION framework. For an extensive description of IRLLS, we refer the reader to the paper in which the approach has been originally proposed^12^. As described in the work of Collier et al.^12^, the IRLLS pipeline starts by checking whether the estimates obtained from an initial WLLS model fit satisfy a GOF criterion in which the following reduced $\chi ^{2}$-statistic ($\chi ^{2}_{red}$) is evaluated:

8 $$\begin{aligned} \chi ^{2}_{red}=\frac{1}{\nu }\sum _{i=1}^{N_{\text {DWI}}} \frac{e_i^2}{\hat{\sigma }^2}{,} \end{aligned}$$

with *ν* the degrees of freedom given by $N_{\text {DWI}} - N_{par}$ and $e_i$ the residuals of the model fitting in the non-linear space, i.e., $e_i=\tilde{S}_i(b,\boldsymbol{g})-\text {exp}(\boldsymbol{b}_{i}^T\hat{\boldsymbol{\theta }}_{\text {WLLS},i})$.

In Eq. (8), $\hat{\sigma }$ represents the standard deviation of the error terms, estimated as:

9 $$\begin{aligned} \hat{\sigma }_{k}=1.4826\sqrt{\frac{N_{\text {DWI}}}{\nu }}\text {med}_{i}(|w_{ii}e_{i}^{*}-\text {med}_{j}(w_{jj}e_{j}^{*})|){,} \end{aligned}$$

where the weights $w_{ii}$ and the residuals in the linear space $e_{i}^{*}$, given by $y_i-\boldsymbol{b}_{i}^T\hat{\boldsymbol{\theta }}_{\text {WLLS},i}$, are obtained from the initial WLLS DKI model fit. Equation (9) is evaluated voxel-wise for $k = 1,..., N_{\text {vox}}$, after which the final estimate of the noise standard deviation is derived as the median across all voxels: $\hat{\sigma }=\text {med}_{k}(\hat{\sigma }_{k})$.

By the central limit theorem, for a large number of observations and in the absence of outliers, the distribution of $\chi ^{2}_{red}$ can be well approximated by a normal distribution with mean *μ =1* and variance $\sigma ^{2}=2/\nu$; therefore, the GOF criterion is considered satisfied if $\chi ^{2}_{red}$ lies within three standard deviations from the mean of the distribution, which corresponds with a 99.7% confidence level. Mathematically, a successful GOF check implies that $\chi ^{2}_{red} \in [1-3\sqrt{2/\nu }, 1+3\sqrt{2/\nu }]$, i.e., $|\chi ^{2}_{red}-1|<3\sqrt{2/\nu }$. If the GOF criterion is satisfied, no additional steps are performed and the initial WLLS estimates are accepted as the final result for the specific voxel under analysis. Differently, every time the GOF step is not satisfied, the framework continues with an iterative re-weigthing procedure and subsequent outlier detection and removal.

In the iterative re-weighting procedure, the Geman-McClure loss function is minimized by means of WLLS and the residuals of the model fit are adjusted at each iteration until convergence is reached or the maximum number of iterations is exceeded. A general expression of the Geman Mc-Clure (GM) loss is given by^26,27^:

10 $$\begin{aligned} \rho (x,k,c)=\frac{\frac{k}{2}(\frac{x}{c})^2}{k+(\frac{x}{c})^2}, \end{aligned}$$

with *k* a constant shape parameter that controls the robustness of the loss and *c* a scale parameter related to the size of the quadratic bowl near *x=0* which can be either fixed or estimated at each iteration. The corresponding GM weighting function is given by the first derivative of *ρ (x,k,c)* divided by *x*:

11 $$\begin{aligned} w_\text {GM}(x,k,c)= \frac{1}{x}\frac{\partial {\rho (x,k,c)}}{\partial {x}} = \frac{\varphi (x,k,c)}{x}=\frac{k^2}{c^2(k+(\frac{x}{c})^2)^2}. \end{aligned}$$

In IRLLS, *x* is given by the residuals in the linear space ($e^*$), *k* is set to 1 and *c* is an estimate of the standard deviation of the *i*-th error term in the linear space, iteratively updated as:

12 $$\begin{aligned} c_{i} = \frac{1.4826\sqrt{\frac{N_{\text {DWI}}}{\nu }}\text {med}_{i}(|e_{i}-\text {med}_{j}(e_{j})|)}{\hat{S}_{i}}, \end{aligned}$$

where the factor $1/\hat{S}_{i}$—with $\hat{S}_{i}$ the *i*-th model prediction—is included to account for the heteroscedasticity of the log-transformed data. An overview of the derivation of the weighting functions for conventional and robust estimators is presented in the Appendix within the Supplementary Material. The iterative re-weighting is repeated until a convergence criterion is met or the maximum number of iterations (set to 100 throughout the paper) is exceeded. As a convergence criterion, if the $L^2$-norm of the difference between the estimated parameter vector at the last two iterations ($||\hat{\boldsymbol{\theta }_l} - \hat{\boldsymbol{\theta }}_{l-1}||_2$, with *l* indexing the current step) is smaller than 0.1% of the $L^2$-norm of $\hat{\boldsymbol{\theta }_l}$, then the iterative procedure stops and the final outlier rejection step is performed.

The outlier detection step exploits the natural skewness of the data distribution through a *two-eyes* approach: for negative residuals the outlier detection is performed in the linear space while for positive residuals, outliers are identified in the non-linear space. Additionally, to mitigate the effect of unequal variances the residuals are *studentized* as

13 $$\begin{aligned} t_{i}^* = \frac{e_i^*\hat{S}_i}{\hat{\sigma }\sqrt{1-h_{ii}}}, \end{aligned}$$

in the linear space and as

14 $$\begin{aligned} t_{i} = \frac{e_i}{\hat{\sigma }\sqrt{1-h_{ii}}}, \end{aligned}$$

in the non-linear space. In Eqs. (13) and (14), $h_{ii}$ represents the leverage for the *i*-th residual extracted from the main diagonal of the hat matrix $\boldsymbol{H}$, computed as:

15 $$\begin{aligned} \boldsymbol{H}=\boldsymbol{B}(\boldsymbol{B}^T\boldsymbol{W}_{\text {GM}}\boldsymbol{B})^{-1}\boldsymbol{B}^T\boldsymbol{W}^T_{\text {GM}}, \end{aligned}$$

where $\boldsymbol{W}_{\text {GM}}$ represents the GM weighting matrix from the last iteration of the iterative procedure. The individual data points whose residuals lie beyond a pre-defined confidence interval are excluded from the final WLLS fit according to the following expressions for the linear and the non-linear space, respectively:

16 $$\begin{aligned} e_i^{*}<-\tau \frac{\sqrt{1-h_{ii}}\hat{\sigma }}{\hat{S}_i} \end{aligned}$$

and

17 $$\begin{aligned} e_i>\tau \sqrt{1-h_{ii}}\hat{\sigma }, \end{aligned}$$

where *τ* represents the decision threshold, set to 3 in Collier et al.^12^. Finally, it is important to highlight that data samples with a leverage higher than a certain threshold (0.85 in the current work), if flagged as outliers, are re-integrated in the final parameter estimation routine given their importance for the model fitting.

Compared to IRLLS, a first novelty of ORION consists in the removal of the GOF step, in line with the REKINDLE algorithm, which was specifically designed for DKI analysis^13^. While this choice implies an increase in computation time because all voxels undergo the re-weighting procedure, the results will be less biased by preliminary assumptions that may not always be valid. The higher the number of outliers in the data, the more likely the GOF check will not be satisfied and the re-weighting procedure will be triggered. However, for lower percentages of outliers in the data (1-5% of $N_{\text {DWI}}$), the GOF criterion may still be satisfied for certain voxels thus resulting in the skipping of the iterative re-weighting procedure and, consequently, in a higher number of undetected outliers (false negatives). This result has been shown in simulation experiments in which we analyzed the distribution of $|\chi ^{2}_{red}-1|$ for different percentages of outliers.

A second element of novelty implies a preliminary (i.e., prior to the iterative re-weighting procedure) outlier check performed on the $b_{0}$ images to increase the sensitivity of detecting outliers in the non-diffusion-weighted measurements, known to be significantly important for the model fit. Outliers in the $b_{0}$ images are initially detected based on the scaled median absolute deviation:

18 $$\begin{aligned} |S_{0,p}-\text {med}_{r}(S_{0,r})|>3 \cdot 1.4826 \cdot \text {med}_{q}(|S_{0,q}-\text {med}_{r}(S_{0,r})|), \end{aligned}$$

where the index $p = 1,..., N_{b_{0}}$ runs over the non-diffusion-weighted measurements ($S_{0,p}$). The $b_{0}$ measurements for which the condition in Eq. (18) is true are flagged as outliers and removed from the dataset while the remaining data points undergo the GM re-weighting procedure along with all the DW images. Should all $b_{0}$ samples be rejected by this twofold approach, the measurement with the highest leverage is retained to perform the final fit.

Finally, in ORION a constrained fitting routine is applied to prevent (or at least limit) that the model fit results in negative kurtosis values due to noise and imaging artifacts^25,28^. For this reason, every time a WLLS fit is performed (i.e., initial fit, iterative GM procedure and final fit), the apparent kurtosis coefficient $K_{\text {APP}}$ is constrained to be positive along 60 directions of a densely sampled hemisphere:

19 $$\begin{aligned} K_{\text {APP}}(\boldsymbol{g}) = \sum ^3_{i,j,k,l=1} g_i g_j g_k g_l K_{ijkl} > 0\text {.} \end{aligned}$$

In this study, the constraint in Eq. (19) is applied uniformly across all methods when comparing ORION to other estimation routines (specifically WLLS and IRLLS). In this way, we can objectively assess ORION as an outlier-robust fitting strategy without the influence of constrained versus unconstrained optimization, which would otherwise penalize WLLS and IRLLS, especially at a lower signal-to-noise ratio (SNR)^28^.

### Single-voxel experiments

The purpose of the single-voxel experiments was to compare ORION with IRLLS in terms of outlier detection and statistical parameter estimation performance. To this end, two experiments were designed:

#### Experiment 1

- Aim: To compare the performance of ORION and IRLLS as a function of the outlier percentage (% outliers).
- Description: The data points were corrupted with variable percentages of outliers (0%, 1%, 5%, 10%, 15%, and 20%) and a fixed SNR equal to 20 defined on the non-diffusion-weighted signals.

#### Experiment 2

- Aim: To compare the performance of ORION and IRLLS as a function of the SNR.
- Description: The data points were corrupted with a fixed percentage of outliers (10%) and variable SNRs (from 15 to 40, step size 5) defined on the non-diffusion-weighted signals.

The data for the simulation experiments were generated following the approach presented in the work of Anania et al.^29^. The DKI parameters that served as ground truth (GT), $\boldsymbol{\theta }_{\text {GT}}$, were extracted by fitting the DKI model to a single-voxel DW signal obtained from a two-compartment white matter (WM) model^30^. This model assumes that WM consists of two non-exchanging compartments: the intra- and the extra-axonal space, which are linked through the axonal water fraction (AWF). The eigenvalues for both compartments and the AWF were randomly sampled from uniform distributions, with lower and upper bounds chosen in agreement with values reported in the literature^30,31^. Subsequently, the GT DKI parameters were used as input in Eq. (3) to simulate the final, noise-free single-voxel signal. In line with the work of Tax et al.^13^, the diffusion protocol consisted of 5 $b_{0}$ images and two non-zero *b*-value shells (*b* = 1.2 and 2.5 ms/*μ*m$\vphantom{0}^{2}$) distributed along 60 unique gradient directions. The corresponding GT DKI metrics resulted in a fractional anisotropy (FA) of 0.69, mean diffusivity (MD) of 0.81 *μ*m$\vphantom{0}^{2}$/ms, and mean kurtosis (MK) of 1.24. Furthermore, GT WMTI metrics were derived from the fourth-order kurtosis tensor, yielding an AWF of 0.46, an intra-axonal diffusivity ($D_{a}$) of 1 *μ*m$\vphantom{0}^{2}$/ms, an axial extra-axonal diffusivity ($D_{e,\parallel }$) of 2.09 *μ*m$\vphantom{0}^{2}$/ms, and a radial extra-axonal diffusivity ($D_{e,\perp }$) of 0.77 *μ*m$\vphantom{0}^{2}$/ms.

Within a Monte Carlo framework, the GT DW signal was replicated $N_{rep}=10^{4}$ times and, in each replicate, a randomly selected subset of observations (out of 125) was corrupted by outliers. The data corruption was obtained by multiplying GT signal values by a scaling factor. Two different scenarios were simulated for each experiment: 1) signal dropout with multiplicative factors ranging from 0 (complete signal loss) to 0.75 and 0.25 step size and 2) signal overshoot with multiplicative factors ranging from 1.25 to 2 (double the value of the underlying signal) and 0.25 step size. Finally, noise was added to the outlier-corrupted signals according to a Rician data distribution.

The performance of the outlier detection step was assessed in terms of sensitivity, specificity, and false discovery rate (FDR), computed as:

20 $$\begin{aligned} \text {Sensitivity} = \frac{\text {TP}}{\text {TP+FN}}, \end{aligned}$$

21 $$\begin{aligned} \text {Specificity} = \frac{\text {TN}}{\text {TN+FP}}, \end{aligned}$$

22 $$\begin{aligned} {\text {FDR} = \frac{\text {FP}}{\text {TP+FP}}}, \end{aligned}$$

with TP indicating the true positives, TN the true negatives, FN the false negatives and FP the false positives. For each metric of interest (FA, MD, MK, AWF, $D_{a}$, $D_{e,\parallel }$ and $D_{e,\perp }$), the mean squared error (MSE) was assessed:

23 $$\begin{aligned} \text {MSE} = \frac{1}{N_{rep}} \sum _{i=1}^{N_{rep}} (\hat{m}_{i} - m_{\text {GT}})^2, \end{aligned}$$

where $\hat{m}_{i}$ denotes the *i*-th estimate of a specific DKI/WMTI metric, and $m_{\text {GT}}$ the corresponding GT value.

### Validation on synthetic and in vivo data

Data used in the preparation of this article were obtained from the Alzheimer’s Disease Neuroimaging Initiative (ADNI) database (adni.loni.usc.edu). The ADNI was launched in 2003 as a public-private partnership, led by Principal Investigator Michael W. Weiner, MD. The primary goal of ADNI has been to test whether serial magnetic resonance imaging (MRI), positron emission tomography (PET), other biological markers, and clinical and neuropsychological assessment can be combined to measure the progression of mild cognitive impairment (MCI) and early Alzheimer’s disease (AD).

More specifically, for the purpose of validating the performance of ORION on synthetic and in vivo data, two dMRI datasets were downloaded from the ADNI-3 cohort^32^. Imaging series included in such cohort underwent quality control at Mayo Clinic, and for this research study we focussed on multi-shell dMRI scans labelled as “fair” quality scans with the assumption that such data are more likely to contain intensity outliers compared to “good” quality scans. Among these cases, we selected multi-shell multiband diffusion-weighted images from two MCI patients, aged 72 and 61 years, hereafter referred to as Dataset 1 and Dataset 2, respectively. Visual inspection revealed the presence of clear signal dropouts in Dataset 1 and signal overshoots in Dataset 2. For Dataset 1, imaging was conducted on a 3T Siemens Advanced Prisma scanner. dMRI data were acquired with a 116 *×* 116 acquisition matrix and reconstructed to an isotropic resolution of 2 *×* 2 *×* 2 mm$\vphantom{0}^{3}$, with a repetition time (TR) of 3400 ms and echo time (TE) of 71 ms. 13 $b_0$ images and 114 diffusion-weighted images were acquired in total: 6 images at *b* = 0.5 ms/*μ*m$\vphantom{0}^{2}$, 48 at *b* = 1 ms/*μ*m$\vphantom{0}^{2}$, and 60 at *b=2* ms/*μ*m$\vphantom{0}^{2}$. For Dataset 2, imaging was performed on a 3T Philips Ingenia Elition X scanner. dMRI data were acquired with a 128 *×* 128 acquisition matrix and reconstructed to an isotropic resolution of 2 *×* 2 *×* 2 mm$\vphantom{0}^{3}$ (TR = 4712.4 ms, TE = 80.4 ms). The acquisition consisted of 9 $b_0$ images and 91 diffusion-weighted images: 6 images at *b* = 0.5 ms/*μ*m$\vphantom{0}^{2}$, 38 at *b* = 1 ms/*μ*m$\vphantom{0}^{2}$, and 47 at *b=2* ms/*μ*m$\vphantom{0}^{2}$.

The selected datasets were first denoised using the Marchenko-Pastur principal component analysis (MPPCA) method^33^ and corrected for Gibbs ringing^34^. Subsequently, motion and eddy current-induced distortions were corrected using the FSL *eddy* toolbox^35^ in two different stages: 1) without outlier correction and 2) with slice-wise outlier replacement by enabling the *--repol* functionality. Throughout this section, we refer to the outputs of these two stages as the preprocessed data and the preprocessed outlier-corrected data, respectively.

 The DKI model was fitted to the preprocessed outlier-corrected data by means of constrained WLLS and the resulting DKI parameter vector ($\boldsymbol{\Theta }_{\text {GT}}$) served as input in Eq. (3) to generate the synthetic outlier-free datasets, with diffusion weighting schemes (*b*-values and gradient directions) in line with the real data acquisition settings. From $\boldsymbol{\Theta }_{\text {GT}}$, we extracted the corresponding GT diffusion tensor and diffusion kurtosis derived metrics, namely FA$\vphantom{0}_{\text {GT}}$, MD$\vphantom{0}_{\text {GT}}$ and MK$\vphantom{0}_{\text {GT}},$ as well as the GT WMTI metrics, i.e., $\text {AWF}_{\text {GT}}$, $D_{a_{\text {GT}}},$ $D_{e,\parallel _{\text {GT}}}$, and $D_{e,\perp _{\text {GT}}}.$ Intensity outliers in synthetic images were realistically introduced according to prior information obtained from FSL *eddy*: for each dataset, a voxel-wise corruption factor was calculated as the element-wise ratio between the preprocessed in vivo data and the preprocessed outlier-corrected in vivo data. As illustrated in Fig. S1 of the Supplementary Material, this corruption factor was element-wise multiplied with the outlier-free synthetic datasets, resulting in realistic outlier-corrupted data. Fig. S1 shows the specific slices selected from a *b = 1* ms/$\upmu \mathrm{m}^{2}$ volume, which were affected by dropout and overshoot in Datasets 1 and 2, respectively. By dividing the mean $S_0$ signal by the average SNR within a WM mask, a constant *σ* was obtained for noise injection according to a Rician distribution. The average SNR was estimated from the noise map extracted using MPPCA, resulting in values of 40 for Dataset 1 and 24 for Dataset 2. Before model fitting, we utilized MPPCA to denoise the synthetic datasets, aiming to create a simulation scenario that closely mirrored the in vivo data processing.

The WM masks were extracted by thresholding the WM fiber orientation distribution function (fODF) estimated via multi-shell multi-tissue (MSMT) constrained spherical deconvolution (CSD)^36^. The performance of three estimation approaches was compared for DKI parameter estimation: WLLS, IRLLS, and ORION. For the selected slices, we computed the error map for each approach and each metric of interest as the difference between estimated and GT values. The error was visually evaluated across the entire slice, and its median absolute value was quantified within the WM mask. Finally, DKI and WMTI parameter estimates were extracted from the preprocessed in vivo datasets using the same fitting routines. We compared the WM distributions of FA, MD, MK, AWF, $D_{a}$, $D_{e,\parallel }$ and $D_{e,\perp }$ obtained from the in vivo data fitting with those derived from the synthetic datasets.

## Results

### Does the GOF step help?

**Fig. 1:**
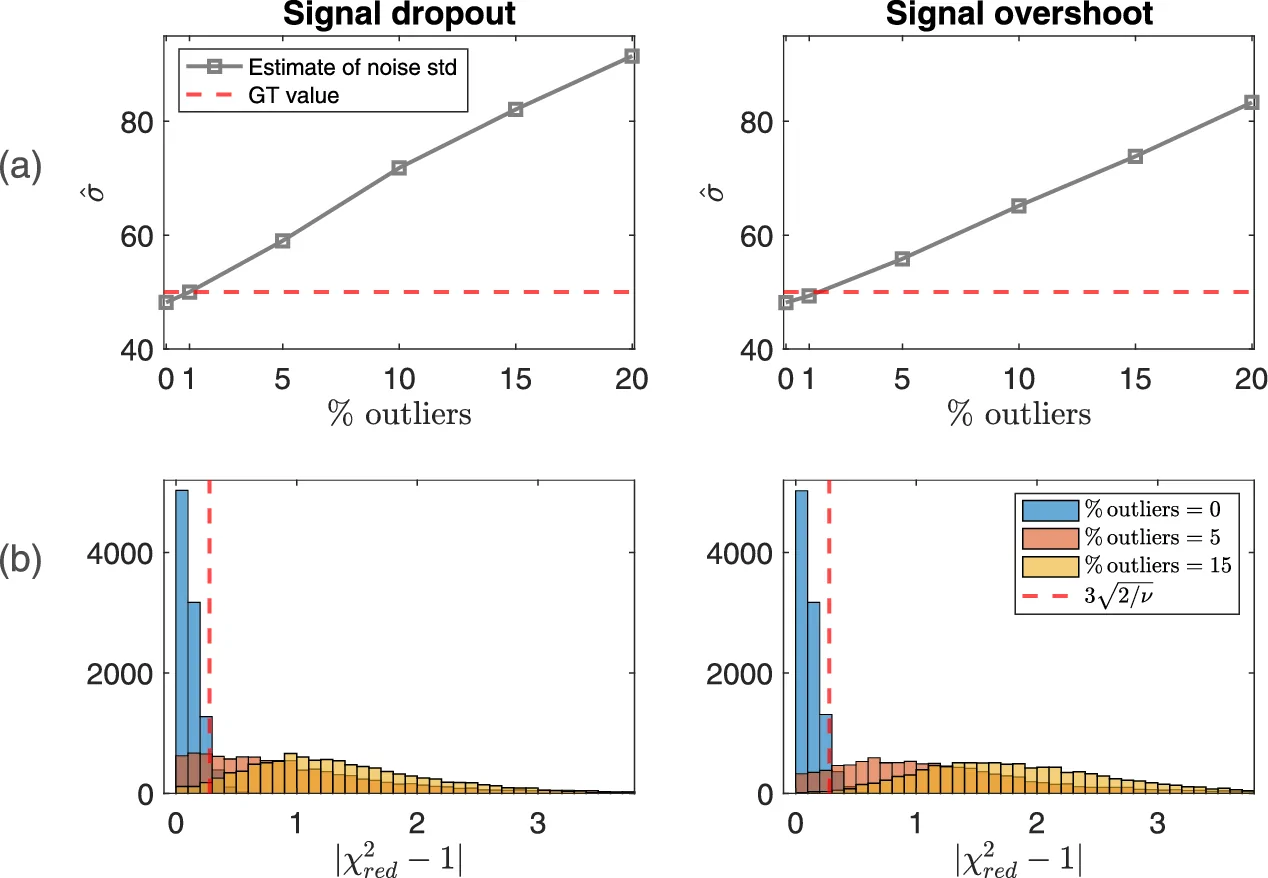
**(a)** Estimates of the noise std ($\hat{\sigma }$) as a function of the percentages of outliers (% outliers): signal dropout scenario (left) and signal overshoot scenario (right). The red dashed line represents the GT value of the noise std according to the simulation settings. **(b)** Distribution of $|\chi ^{2}_{red}-1|$ for three different percentages of outliers (0%, 5% and 15%): signal dropout scenario (left) and signal overshoot scenario (right). The red dashed line represents the threshold value given by $3\sqrt{2/\nu }$: if $|\chi ^{2}_{red}-1|$ is lower than this threshold, then the GOF criterion is satisfied with a 99.7% confidence level; *ν* is given by $N_{\text {DWI}}-N_{par}$ and is equal to 103 (125 - 22) according to the simulation settings.

To assess how often the GOF criterion is satisfied, single-voxel diffusion-weighted signals were generated for different percentages of outliers: 0%, 1%, 5%, 10%, 15% and 20%. Both the signal dropout and the signal overshoot scenarios were considered and noise was introduced according to a Rician data distribution with an SNR of 20. Given a non-diffusion-weighted signal $S_{0} = 10^3$, the GT value of the noise standard deviation (std) is obtained as $S_{0}$/SNR, resulting in a value of 50. Figure 1 shows (a) the deviation of the estimated noise standard deviation from the ground truth value for the simulated percentages of outliers, and (b) the distribution of $|\chi ^{2}_{\text {red}} - 1|$ for three specific outlier percentages (0%, 5%, and 15%).

For lower percentages of outliers, namely 0% and 1%, the estimates of the noise std are close to the actual GT value confirming that the expression in Eq. (9) provides a reliable noise estimation. On the other hand, for higher % outliers, the noise estimates start deviating from the GT value and, as expected, this deviation increases as the percentage of outliers in the data increases. From Fig. 1b, we can highlight that when no outliers are introduced in the data, the GOF criterion is satisfied in 93% of the repetitions. When % outliers = 5, the GOF step is less frequently satisfied, namely in 18.06% of the cases for the signal dropout scenario and in 9.55% of the cases for the signal overshoot simulations. By further increasing the percentage of outliers to 15%, a successful GOF criterion is observed in 3.64% and 0.61% of the repetitions for the dropout and overshoot scenario, respectively.

The results of this simulation experiment highlight that the GOF step may not be beneficial since, to a certain extent, it is also satisfied for outlier-corrupted data. When data containing outliers satisfy the GOF criterion, they do not undergo the iterative re-weighting procedure and, consequently, the WLLS tensor fit will provide suboptimal parameter estimates.

### Single-voxel experiments

#### Performance as a function of the percentage of outliers

**Fig. 2:**
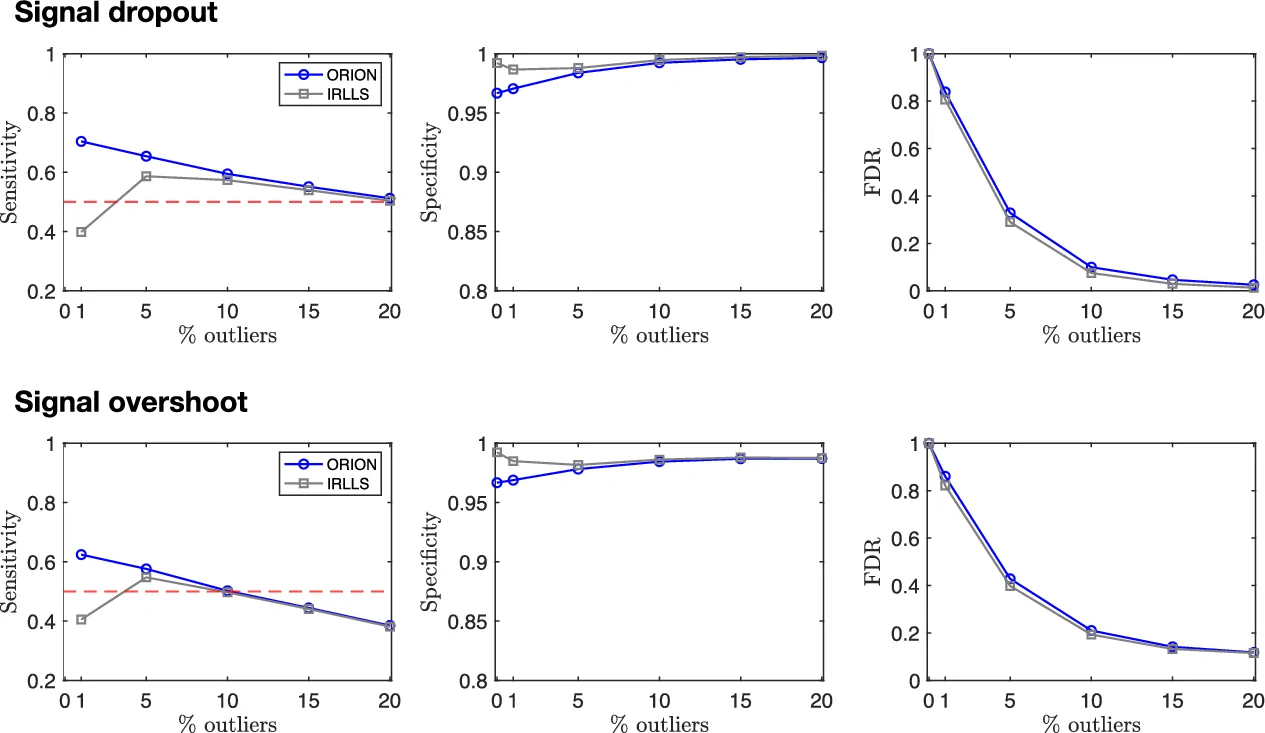
Sensitivity, specificity, and FDR of the outlier detection step as a function of the percentage of outliers (% outliers) for IRLLS and ORION. The two frameworks are compared for both outliers manifesting as signal losses (top row) and outliers manifesting as signal overshoots (bottom row). The red dashed line corresponds to a 50% sensitivity.

The sensitivity, specificity and FDR of the outlier detection step were assessed for different percentages of outliers and for both the signal dropout and the signal overshoot scenario. The SNR, defined on the $b_0$ signal, was fixed at 20, and the results obtained using the IRLLS framework and ORION are presented in Fig. 2.

**Fig. 3:**
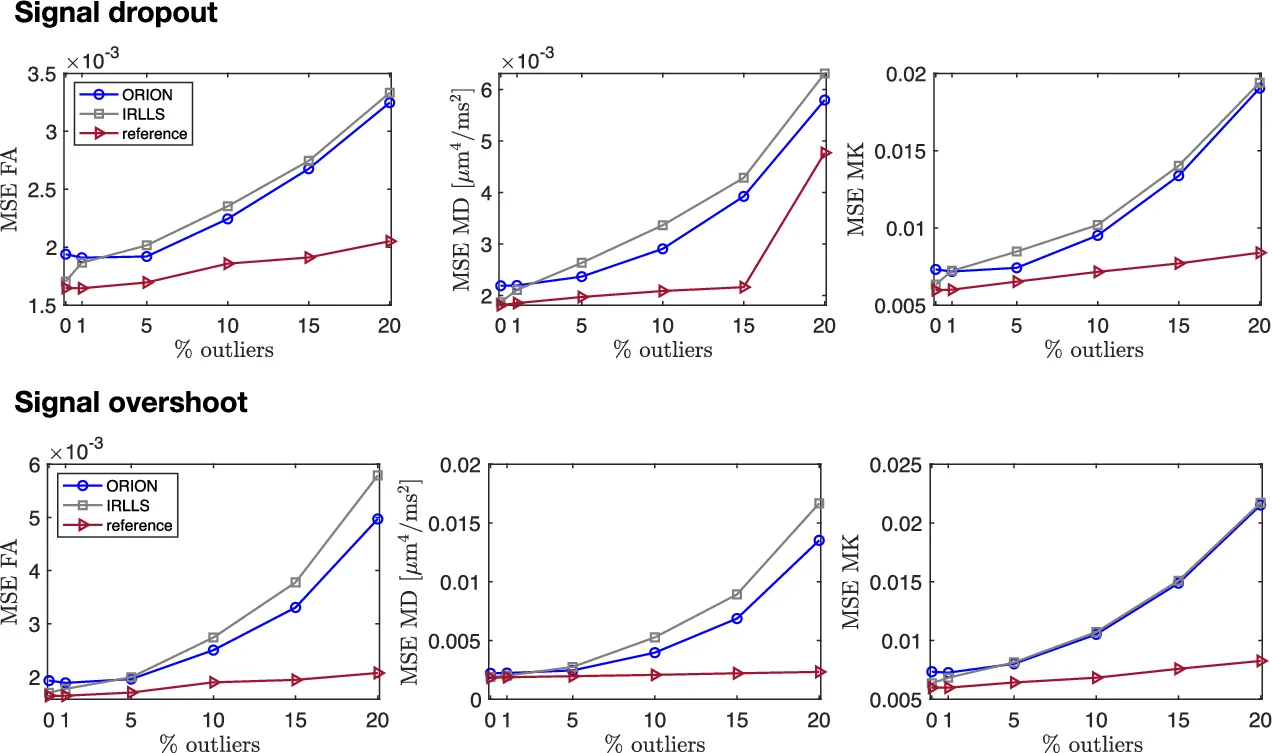
Mean squared error (MSE) of FA, MD and MK as a function of the percentage of outliers (% outliers) for IRLLS, ORION and the “reference” approach. The different methods are compared for both outliers manifesting as signal losses (top row) and outliers manifesting as signal overshoots (bottom row).

Figure 2 shows that ORION outperforms IRLLS in terms of sensitivity of the outlier detection step at the cost of a slight loss in specificity and FDR for both signal dropouts and signal overshoots. The framework is more sensitive at detecting signal losses compared to signal overshoots: in the case of signal dropout, ORION results in sensitivity values higher than 50% with up to 20% of outliers in the data, whereas the sensitivity drops below 50% for percentages higher than 10% in the case of signal overshoot. In Fig. 3, the MSE of FA, MD and MK is reported for IRLLS, ORION and for a hypothetical perfect outlier detection method (100% sensitivity and specificity), referred as the “reference” approach (i.e., the best imaginable solution) throughout the manuscript. ORION outperforms IRLLS starting from 5% of outliers in the data for all three metrics when considering signal dropouts and for FA and MD when considering signal overshoots. Supplementary Fig. S2 shows that ORION outperforms IRLLS for $D_{a}$, $D_{e,\parallel }$ and $D_{e,\perp }$ in signal dropout scenarios, starting at a 5% outlier rate (top row). In contrast, in signal overshoot scenarios, the main benefits of ORION over IRLLS are observed for $D_{e,\parallel }$ and $D_{e,\perp }$ starting from 10% outliers (bottom row).

#### Performance as a function of the SNR

**Fig. 4:**
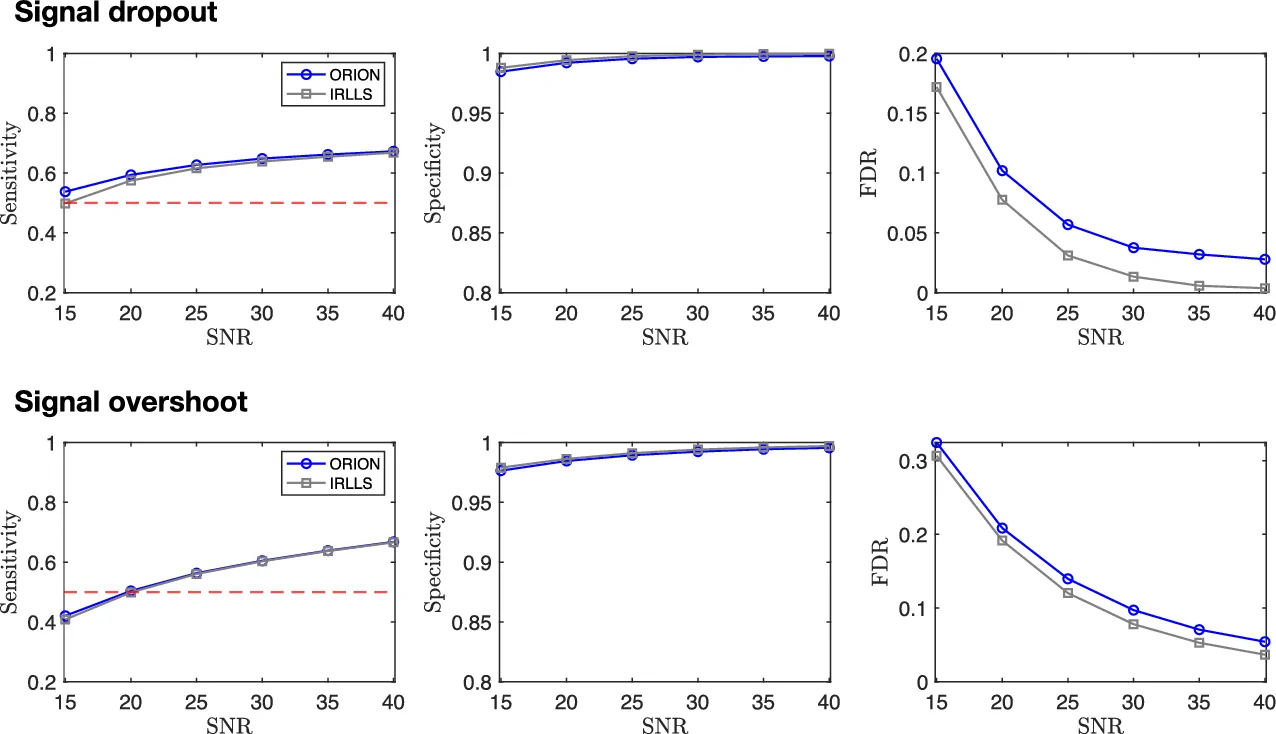
Sensitivity, specificity, and false discovery rate (FDR) of the outlier detection step as a function of the SNR for IRLLS and ORION. The two frameworks are compared for both outliers manifesting as signal losses (top row) and outliers manifesting as signal overshoots (bottom row). The red dashed line corresponds to a 50% sensitivity.

In this experiment, the performance of the outlier detection step was assessed in terms of sensitivity, specificity and FDR for different SNRs, i.e., [15, 20, ..., 35, 40]. Both the signal dropout and the signal overshoot scenarios were considered and the percentage of outliers was fixed at 10%. Figure 4 shows that the sensitivity of ORION is higher than that of IRLLS for SNR values up to 25 in the case of signal dropout. On the other hand, for signal overshoots, no notable differences are observed.

**Fig. 5:**
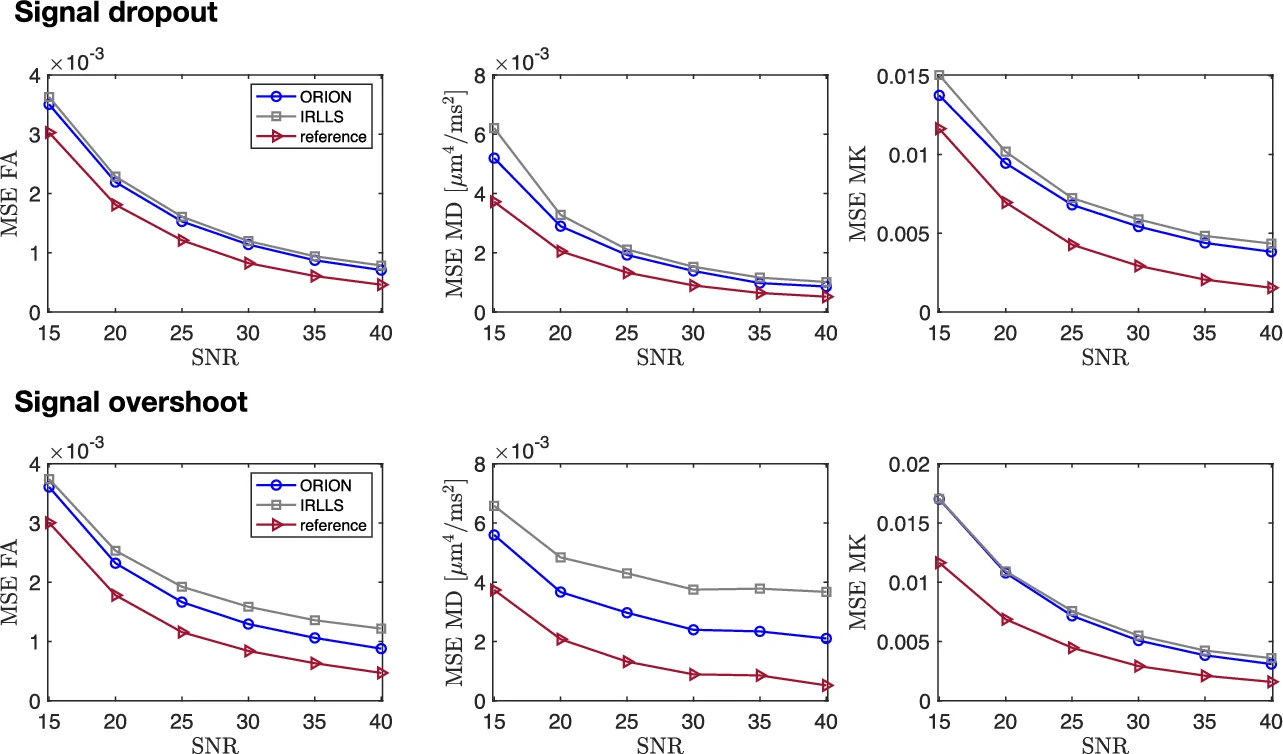
Mean squared error (MSE) of FA, MD and MK as a function of the SNR for IRLLS, ORION and the “reference” approach. The different methods are compared for both outliers manifesting as signal losses (top row) and outliers manifesting as signal overshoots (bottom row).

The performance in terms of statistical parameter estimation is reported in Fig. 5: ORION better approaches the “reference” MSE curve compared to IRLLS for the three metrics under assessment, except for MK in the case of signal overshoots, where both frameworks exhibit almost overlapping performance. For WMTI metrics, ORION outperforms IRLLS for $D_{a}$, $D_{e,\parallel }$, and $D_{e,\perp }$ under signal dropout conditions, particularly at lower SNRs (Supplementary Fig. S3, top row). In the signal overshoot scenario, the two methods show comparable performance overall, except for $D_{e,\perp }$, where ORION consistently outperforms IRLLS across all SNRs (Fig. S3, bottom row).

### Validation on synthetic and in vivo data

**Fig. 6:**
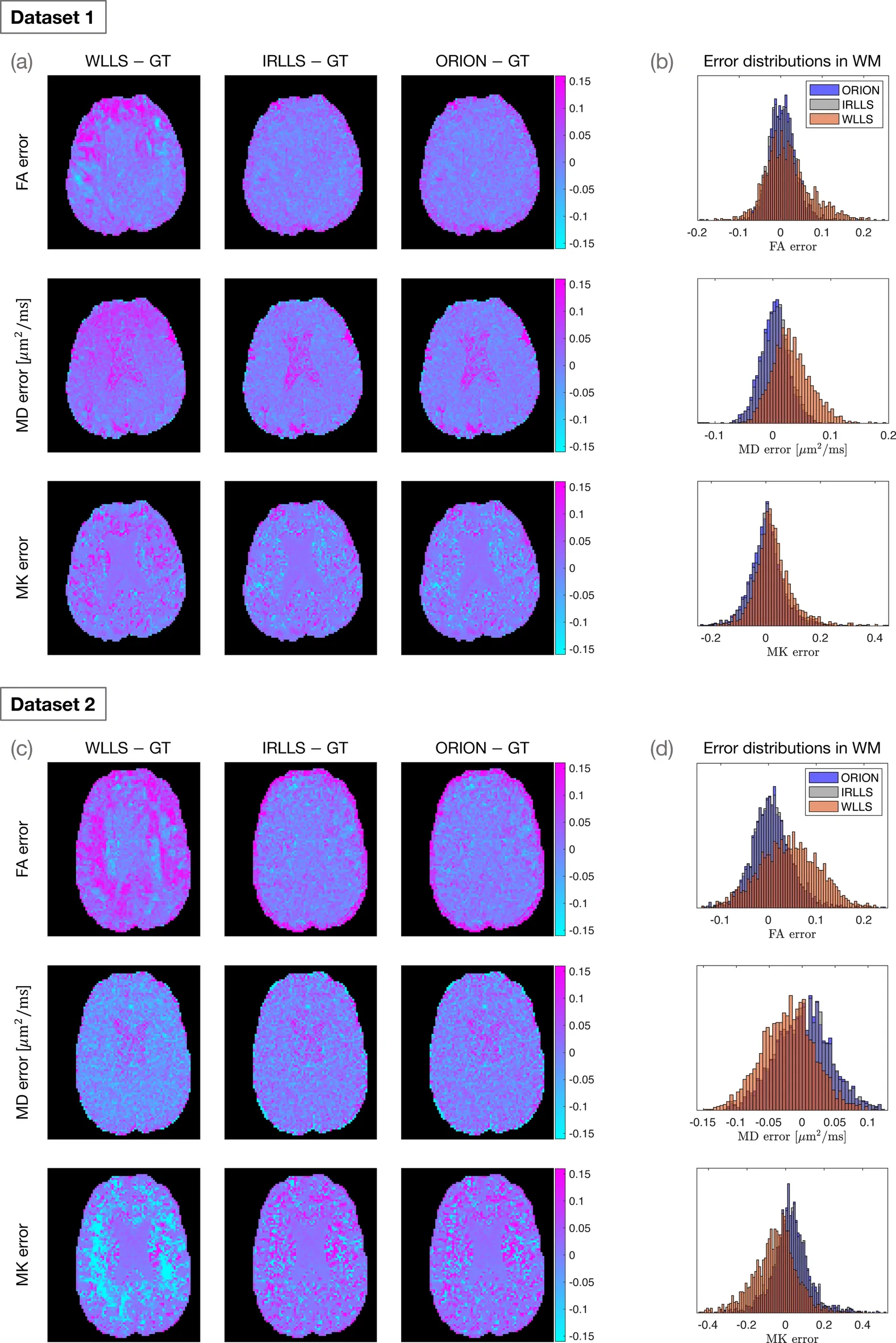
Performance comparison on Dataset 1 (**a**, **b**) and Dataset 2 (**c**, **d**). (**a**, **c**) Voxel-wise error maps for FA, MD, and MK across an outlier-corrupted axial slice of the synthetic datasets. The maps are visually compared for the three parameter estimation approaches under assessment: WLLS (left), IRLLS (middle), and ORION (right). (**b**, **d**) Error distributions for the same metrics and the same slice restricted to white matter (WM). The distributions are reported for the conventional WLLS approach and for the two outlier-robust approaches, IRLLS and ORION.

In Fig. 6a–c, visual error maps of FA, MD, and MK are shown for two outlier-corrupted slices selected from Datasets 1 and 2, respectively. For each metric, the errors are computed as the voxel-wise difference between the estimated value and the corresponding GT value. Across both datasets and all three metrics, the use of an outlier-robust strategy, namely IRLLS or ORION, results in errors closer to 0 compared to the conventional WLLS estimation approach. Since it is difficult to visually compare IRLLS and ORION, and given that these techniques are designed to identify and reject sporadic, direction-specific outliers in brain parenchyma (primarily WM), histograms of the error distributions for FA, MD, and MK inside a WM mask are reported in Fig. 6b–d. The WM error distributions are quantitatively summarized as the median absolute errors in Table 1, where the three estimation methods (WLLS, IRLLS, and ORION) are statistically compared using a two-sided Wilcoxon signed-rank test with Bonferroni correction.

The results in Table 1 show that IRLLS and ORION achieve significantly lower errors than WLLS in all cases. Between IRLLS and ORION, there are no statistically significant differences for Dataset 2, whereas for Dataset 1, differences are significant for FA and MD, but not for MK.

**Table 1:** Median absolute error of FA, MD, and MK within a WM mask for Datasets 1 and 2.

Dataset	Metric	WLLS	IRLLS	ORION
Dataset 1	$\text {med}_{i}(|\widehat{\text {FA}}_{i} - \text {FA}_{\text {GT},i}|)$	0.0314	0.0213$\vphantom{0}^{a}$	0.0202$\vphantom{0}^{a,b}$
	$\text {med}_{i}(|\widehat{\text {MD}}_{i} - \text {MD}_{\text {GT},i}|)$ [*μ*m$\vphantom{0}^{2}$/ms]	0.0300	0.0164$\vphantom{0}^{a}$	0.0155$\vphantom{0}^{a,b}$
	$\text {med}_{i}(|\widehat{\text {MK}}_{i} - \text {MK}_{\text {GT},i}|)$	0.0383	0.0358$\vphantom{0}^{a}$	0.0358$\vphantom{0}^{a}$
Dataset 2	$\text {med}_{i}(|\widehat{\text {FA}}_{i} - \text {FA}_{\text {GT},i}|)$	0.0538	0.0259$\vphantom{0}^{a}$	0.0259$\vphantom{0}^{a}$
	$\text {med}_{i}(|\widehat{\text {MD}}_{i} - \text {MD}_{\text {GT},i}|)$ [*μ*m$\vphantom{0}^{2}$/ms]	0.0303	0.0280$\vphantom{0}^{a}$	0.0280$\vphantom{0}^{a}$
	$\text {med}_{i}(|\widehat{\text {MK}}_{i} - \text {MK}_{\text {GT},i}|)$	0.0748	0.0564$\vphantom{0}^{a}$	0.0567$\vphantom{0}^{a}$

The median is computed across $N_S$ voxels from the selected axial slices ($i = 1, \ldots , N_S$), where $N_S = 1896$ for Dataset 1 and $N_S = 2149$ for Dataset 2. For each dataset, statistical significance was assessed using a two-sided Wilcoxon signed-rank test with Bonferroni correction for multiple comparisons (*α = 0.05/9*).

$\vphantom{0}^{a}$ Significant difference compared to WLLS (*p < 0.0056*).

$\vphantom{0}^{b}$ Significant difference compared to IRLLS (*p < 0.0056*).

**Fig. 7:**
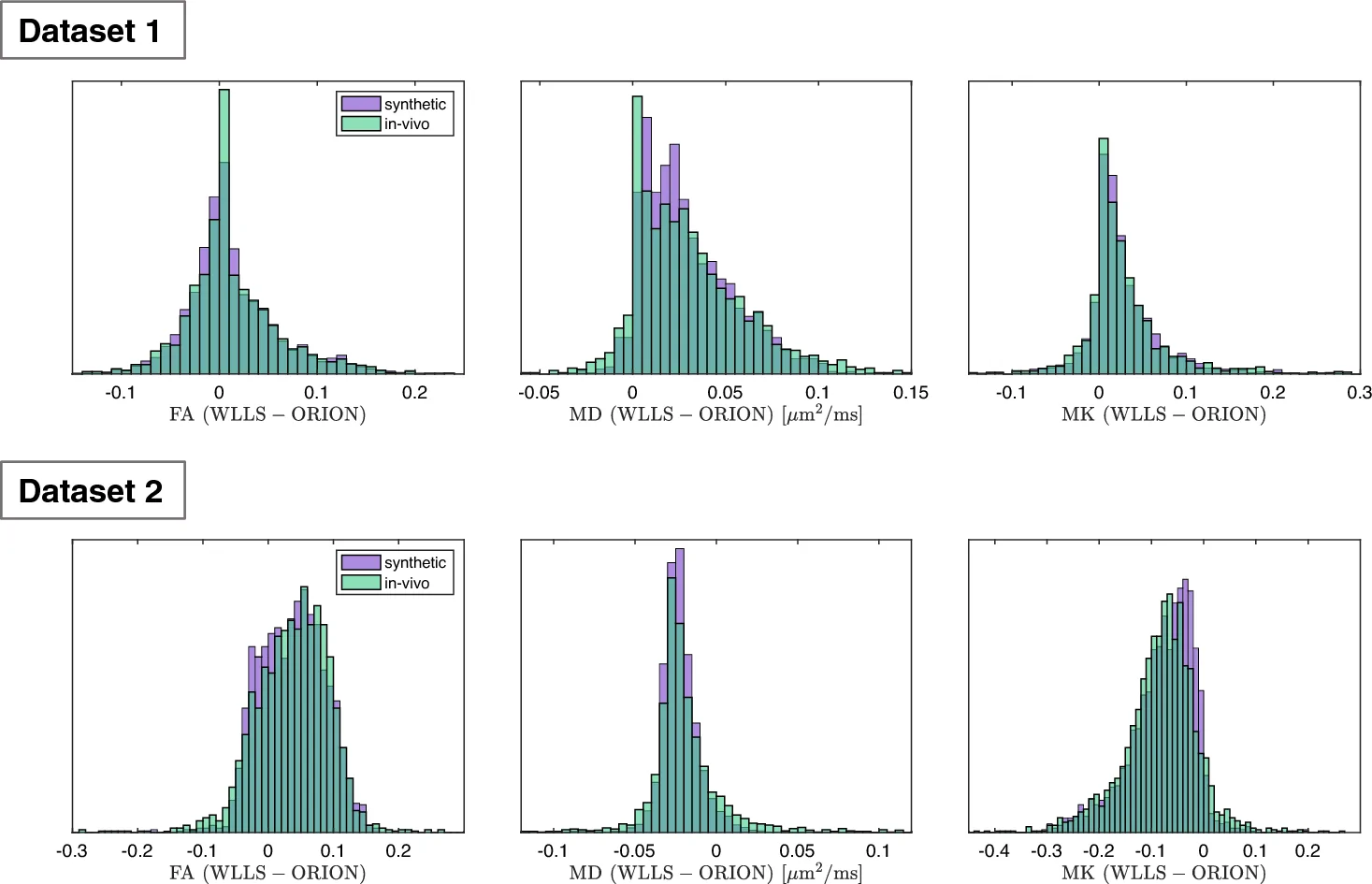
Distributions of the difference between WLLS and ORION estimates of FA, MD, and MK within a WM mask. Results are shown for an outlier-corrupted axial slice of both synthetic and in vivo datasets: Dataset 1 (top row) and Dataset 2 (bottom row).

Figure 7 displays the differences between WLLS and ORION WM estimates for FA, MD, and MK. These differences are evaluated across the identified outlier-corrupted slices for both the synthetic and in vivo datasets. The distributions confirm that the in vivo estimates are in line with the synthetic data trends. Results for WMTI metrics on synthetic and in vivo data are provided in the Supplementary Material (Figs. S4-S6 and Table S1). For the visual error maps and WM error distributions, we refer the reader to Figs. S4 and S5. Table S1 indicates that: 1) ORION and IRLLS significantly outperform WLLS for the four WMTI metrics; 2) ORION achieves significantly lower errors than IRLLS for AWF, $D_{e,\parallel }$, and $D_{e,\perp }$ in Dataset 1; and 3) no statistically significant differences exist between ORION and IRLLS in Dataset 2. Lastly, the comparison between synthetic and in vivo WMTI estimates (see Fig. S6) suggests a strong agreement between their respective distributions.

## Discussion

### Motivation: a framework for outlier-robust DKI parameter estimation

The presence of intensity outliers in DW images is a well-known problem in the MRI community. Such imaging artifacts are mainly due to motion-related processes such as within-volume motion, cardiac pulsation and respiratory effects, and the intrinsically low SNR of dMRI acquisitions, especially at higher *b*-values, makes outlier detection particularly challenging. Motion-induced outliers typically manifest as regional or slice-wise signal losses, thus resulting in darker areas within the image or volume. However, intensity outliers may also translate into signal spikes, as it happens when spin-history effects occur^37^, and may originate from causes other than motion, e.g., hardware-related instabilities. The impact of including artifactual data points into model fitting routines is seen as biased or less precise estimates of the brain tissue microstructural properties, potentially affecting the conclusions drawn in studies involving both control and patient populations. For these reasons, several outlier correction strategies have been proposed in the scientific literature, mainly as voxel-wise or slice-wise techniques and as model-dependent or model-independent solutions. In the current work we present a voxel-wise model-dependent framework, coined ORION, for outlier-robust DKI parameter estimation for two main reasons: 1) although slice-wise approaches have shown promising results^15,16,18–20^, informative non-outlier data points may still be present in slices flagged as outliers; and 2) while model-independent frameworks are more generalizable, the DTI and DKI models still represent the most popular choices to extract quantitative parameters from dMRI datasets. In line with REKINDLE^13^, an approach specifically designed for DKI acquisitions, ORION introduces a simple yet effective modification to the IRLLS technique^12^ by removing the GOF step. In single-voxel simulation experiments, we have shown that this modification is beneficial both in terms of sensitivity of the outlier detection step (cf. Figs. 2 and 4) and in terms of statistical parameter estimation (cf. Figs. 3, 5, S2, and S3). In addition to the removal of the GOF criterion, another element of novelty of ORION is the preliminary outlier rejection on the $b_{0}$ images. Such images play a significant role in the model fit; consequently, $b_{0}$ outliers need to be properly handled, especially since previously proposed voxel-wise methods did not account for the introduction of outlier data points in the non-diffusion-weighted shell^6,8–10,12,13^ or did not specify it^23^. Though the iterative re-weighting procedure of the IRLLS framework, as originally proposed, targets images acquired with any *b*-value, we have found that the sensitivity of detecting artifactual $b_{0}$ observations is further increased when including an extra step that excludes outliers based on the scaled median absolute deviation (cf. Eq. (18)) prior to the iterative loop.

Another difference between our framework and previous strategies^6,12,13^ is given by the introduction of constraints into the fitting routine both in the GM procedure and in the final fit. The benefits of constrained estimation in the context of outlier detection were previously reported in the work of Maximov et al.^9^, where a positivity constraint was applied to prevent the generation of negative eigenvalues when reconstructing diffusion tensors. Considering that DKI metrics are more sensitive to noise and artifacts compared to DTI parameters, we have constrained the minimization procedure by ensuring positiveness of the apparent kurtosis coefficient in the directions of a densely sampled hemisphere. Posing this constraint provides more accurate and precise parameter estimates, particularly at lower SNR values, with a loss in computational efficiency as the only drawback.

### Performance across synthetic and in vivo datasets

Our synthetic data simulations show that ORION outperforms the conventional, non-outlier-robust WLLS approach in terms of accuracy and precision for both standard DKI metrics (FA, MD and MK) and biophysical parameters (AWF, $D_{a}$, $D_{e,\parallel }$ and $D_{e,\perp }$) in white matter regions affected by intensity outliers. Compared to IRLLS, ORION leads to significantly lower errors in signal dropout scenarios (Dataset 1) for most metrics: FA, MD, AWF, $D_{e,\parallel }$, and $D_{e,\perp }$ (cf. Table 1 and Table S1). No statistically significant differences are observed for the signal overshoot scenario (Dataset 2), which is not unexpected, as the single-voxel experiments (cf. Figs. 2 and 4) indicate that ORION is more sensitive than IRLLS to signal dropouts, with a less pronounced advantage for overshoots. Overall, the superiority of ORION in addressing signal dropouts, its non-inferiority for signal overshoots, and the higher likelihood of signal losses in real-world acquisitions motivate its selection over IRLLS.

The whole brain simulations presented in this study were designed with the intention to identify some trends in the estimated metrics that could reflect the behavior observed in in vivo data. While the metrics extracted from the in vivo datasets show strong agreement to the synthetic results, differences in their respective distributions can still be observed (cf. Fig. 7 and Fig. S6). There may be several reasons for which the real data results do no entirely match our simulations. The most important aspect lies in the lack of ground truth: while in simulations we perfectly know which data points represent outlier observations, this is not the case in the real data scenario. Even though the dMRI scans included in the analysis are labelled as “fair” quality data, we have no prior information on the actual presence and distribution of outliers in these images. Another important difference between simulations and real data analysis is given by the preprocessing steps: as opposed to the synthetic datasets, which are fed into the fitting algorithms immediately after denoising, the real data undergo not only denoising but also Gibbs ringing removal, along with motion and distortion correction prior to model fitting. These additional correction blocks may alter the original distribution of outliers.

### Selection of the decision threshold

Outlier-robust strategies that reject artifactual observations imply selecting a decision threshold based on which a binary decision can be made. The adoption of binary rules to identify outliers is common practice in diffusion MRI, not only for voxel-wise approaches such as ORION, IRLLS, and REKINDLE, but also for slice-wise strategies such as the *--repol* functionality implemented in FSL *eddy*^18^. However, choosing a binary decision threshold necessitates a trade-off between sensitivity and specificity and its selection can be user-dependent, which represents a limitation.

In this study, the decision threshold *τ* was set to 3 for single-voxel experiments and adjusted to 4 for whole-brain synthetic and in vivo datasets. Such choice was initially guided by visual inspection, as the outlier maps extracted from the synthetic and in vivo datasets revealed a higher false positive rate with *τ =3* compared to *τ =4*, especially within $b_0$ images where signal variance is naturally higher. Beyond visual assessment, our threshold selection aligns with previous literature and existing software implementations. Specifically, a threshold of 3 has been established to offer a good balance between sensitivity and specificity in single-voxel simulations^6,12,13^, whereas a threshold of 4 was employed by Koch et al.^23^ to reduce false positives in in vivo clinical data. Furthermore, the default *--ol_nstd* parameter in FSL *eddy*^18^, which defines the number of standard deviations a slice must deviate to qualify as an outlier, is likewise set to 4. Finally, while a threshold of 3 was suggested for REKINDLE based on simulation findings, its default value is more conservatively set to 6 in the open-source implementation^13,19^.

### Applicability to other diffusion models

Considering that IRLLS was proposed for the DTI model, we have performed single-voxel experiments to confirm that the benefits of ORION also hold in the case of DTI signal representation. A single-voxel DW signal was generated as described in the methods but only using the 6 independent diffusion tensor elements of $\boldsymbol{\theta }_{\text {GT}}$ and a diffusion protocol suitable for DTI acquisitions, consisting of 5 *b* = 0 ms/*μ*m$\vphantom{0}^{2}$ images and 30 *b* = 1 ms/*μ*m$\vphantom{0}^{2}$ images. The data points were corrupted with variable percentages of outliers (0%, 5%, 10%, 15% and 20%) and noise was introduced with a fixed SNR equal to 20 defined on the $b_{0}$ signal following a Rician data distribution. Both signal dropouts and signal overshoots were simulated in line with the DKI experiments. The results are reported in the Supplementary Material and match the findings obtained in the DKI single-voxel experiments: ORION outperforms IRLLS both in terms of sensitivity of the outlier detection step (see Supplementary Fig. S7) and in terms of the MSE of FA and MD (see Supplementary Fig. S8).

Regarding the applicability of ORION to other diffusion models beyond DTI and DKI, it is important to emphasize that the framework is currently tailored to models that 1) allow for a linear or linearized estimation step and 2) produce residual distributions that are statistically compatible with the *two-eyes* approach design. Even when theoretical compatibility is ensured, extending ORION to other available diffusion models requires dedicated investigation and potential technical or methodological adjustments. As it stands, the validation of ORION on DTI and DKI, including WMTI metrics, supports its use in well-defined applications.

Looking forward, a promising path for extending ORION would be to extract outlier-robust WMTI parameters by fitting the axisymmetric DKI model as proposed by Jespersen et al.^38^. By assuming an axially symmetric fODF around an unknown symmetry axis, this approach reduces the number of independent parameters to be estimated from 22 to 8. However, incorporating such a parameterization into ORION would require redesigning the algorithm from a weighted linear to a fully non-linear variant.

### Limitations and future perspectives

Our study comes with some limitations, the main one being the elevated FDR at low *b*-values. In simulations, we showed that the FDR decreases for higher percentages of outliers in the data (cf. Fig. 2) and at higher SNRs (cf. Fig. 4). Therefore, a loss in performance may be expected in datasets with low percentages of outliers or in datasets characterized by poor SNR. In future work, ORION could be further validated on datasets that are severely corrupted by outliers to better assess its performance relative to conventional estimation routines. Moreover, we could design test-retest DKI studies similar to existing ones^39–41^, but explicitly instruct participants to move during acquisitions, thereby inducing outliers and enabling further validation of the algorithm. A second limitation consists in the need for fine-tuning the decision threshold depending on the presence of outliers in the input data, which is also quite difficult to assess purely based on visual inspection. This limitation could be overcome by removing the binary rejection component of the framework and considering alternative approaches such as signal imputation^18,23^ or downweighting^19^. In this context, Sairanen and Andersson^5^ recommend outlier replacement over downweighting for least-squares-based estimation, while noting that downweighting may provide more accurate parameter estimates in applications such as probabilistic tractography.

The findings of our study can form the basis for interesting future efforts. A first avenue would be to compare the performance of our strategy with the most employed voxel-wise approaches for outlier detection in dMRI. A second promising direction is to explore whether the performance of ORION can be enhanced by combining it with slice-wise outlier detection strategies. Indeed, in ORION’s current implementation, spatial correlations of outliers are ignored, while in practice such correlations are likely to exist. Slice-wise outlier detection methods^15–20^ could be used to inform ORION about likely correlations within a slice, opening opportunities for future research.

## Conclusions

In conclusion, a voxel-wise framework for outlier-robust DTI and DKI parameter estimation has been presented. Such framework, named ORION, employs constrained WLLS estimation and effectively detects and rejects intensity outliers that produce either signal losses or signal spikes. ORION accounts for and corrects outliers in both diffusion-weighted and non-diffusion-weighted ($b_0$) images. In addition, the use of constraints in the fitting routine successfully mitigates the issue of “black voxels” in DKI data, i.e., implausible negative kurtosis estimates typically caused by signal artifacts. Results show that when processing diffusion datasets containing artifactual measurements, the proposed technique provides more accurate and precise parameter estimates compared to conventional estimation approaches.

## Supplementary Information

Supplementary Information.

## Data Availability

The in vivo datasets analyzed in this study were downloaded from the ADNI-3 cohort (adni.loni.usc.edu). The code used for data processing and analysis is available from the corresponding author upon reasonable request.
