# Supplementary material for: The ORION framework for outlier-robust parameter estimation in diffusion kurtosis imaging
Source: Sci Rep. 2026 Aug 13;16:37885. doi: 10.1038/s41598-026-62390-w (PMC13759298; doi:10.1038/s41598-026-62390-w)
Supplement: Supplementary file 1 [file 41598_2026_62390_MOESM1_ESM.pdf]

Supplementary Material
The ORION framework for outlier-robust parameter estimation in diffusion kurtosis imaging

Vincenzo Anania[1,2,*], Ben Jeurissen[1,3], Thibo Billiet[2], Jan Sijbers[1,3], and Arnold J. den Dekker[1,3]

[1]imec - Vision Lab, Department of Physics, University of Antwerp, Antwerp, Belgium
[2]icometrix, Leuven, Belgium
[3]µNEURO Research Centre of Excellence, University of Antwerp, Antwerp, Belgium
[*]vincenzo.anania@uantwerpen.be

Appendix. Derivation of LLS, WLLS and Geman-McClure weighting functions

This appendix summarizes how the weighting functions are obtained for LLS, WLLS, and three variants of the Geman-McClure estimator that have been proposed for outlier-robust dMRI parameter estimation. Table A1 provides, for each estimator, the derivation of its corresponding weighting function, starting from the loss function. For the Geman-McClure estimators, the derivations follow the M-estimation theory, according to Eqs. (10) and (11) of the main text.

Estimator	Loss	Influence	Weight
LLS	$\rho(e_i^*) = \frac{1}{2} e_i^{*2}$	$\varphi(e_i^*) = e_i^*$	$w(e_i^*) = 1$
WLLS	$\rho(e_i^*) = \frac{e_i^{*2}}{2\sigma_i^{*2}}$	$\varphi(e_i^*) = \frac{e_i^*}{\sigma_i^{*2}}$	$w(e_i^*) = \frac{1}{\sigma_i^{*2}}$
Geman-McClure: RESTORE as in Dipy[1]	In Eq. (10) of the main text, we set $x = e_i$, $k = 1$ and $c = 1.4826\,\mathrm{med}_i(\lvert e_i - \mathrm{med}_j(e_j)\rvert)$		
	$\rho(e_i) = \frac{e_i^2}{2(e_i^2+c^2)}$	$\varphi(e_i) = \frac{e_i c^2}{(e_i^2+c^2)^2}$	$w(e_i) = \frac{c^2}{(e_i^2+c^2)^2}$
Geman-McClure: IRLLS[2]	In Eq. (10) of the main text, we set $x = e_i^*$, $k = 1$ and $c = \sigma_i^*$, with $\sigma_i^* = \frac{1.4826\sqrt{\frac{N_{\mathrm{DWI}}}{v}}\,\mathrm{med}_i(\lvert e_i - \mathrm{med}_j(e_j)\rvert)}{\hat{S}_i}$		
	$\rho(e_i^*) = \frac{e_i^{*2}}{2(e_i^{*2}+\sigma_i^{*2})}$	$\varphi(e_i^*) = \frac{e_i^* \sigma_i^{*2}}{(e_i^{*2}+\sigma_i^{*2})^2}$	$w(e_i^*) = \frac{\sigma_i^{*2}}{(e_i^{*2}+\sigma_i^{*2})^2}$
Geman-McClure: REKINDLE[3]	In Eq. (10) of the main text, we set $x = d_i = \frac{e_i^*}{\hat{\sigma}}$, $k = 1$ and $c = 1$, with $\hat{\sigma} = 1.4826\,\mathrm{med}_i(\lvert e_i^* - \mathrm{med}_j(e_j^*)\rvert)$		
	$\rho(d_i) = \frac{d_i^2}{2(d_i^2+1)}$	$\varphi(d_i) = \frac{d_i}{(d_i^2+1)^2}$	$w(d_i) = \frac{1}{(d_i^2+1)^2}$ $\Rightarrow w(e_i^*) = \frac{\hat{\sigma}^4}{(e_i^{*2}+\hat{\sigma}^2)^2}$

Table A1. Derivation of weighting functions for different estimators.

In summary, the weighting functions presented in Table A1 highlight the key differences among the estimators. WLLS, IRLLS, and REKINDLE derive their weights as functions of residuals in the linear space. In WLLS, the weight computation depends on σ_i^*, which is an estimate of the standard deviation of the error terms in the linear space and varies across measurements. In IRLLS, the scale parameter c depends on the i-th measurement through the factor $1/\hat{S}_i$, thus effectively accounting for and mitigating heteroscedasticity. While IRLLS derives weights for unscaled residuals within an iterative WLLS framework, REKINDLE computes weights for residuals scaled by $\hat{\sigma}$ and assumes a unit shape parameter. Finally, in RESTORE, the weights are computed directly in the non-linear space, eliminating the need to explicitly model heteroscedasticity, and the scale parameter c is treated as a constant.

Supplementary Figures and Tables

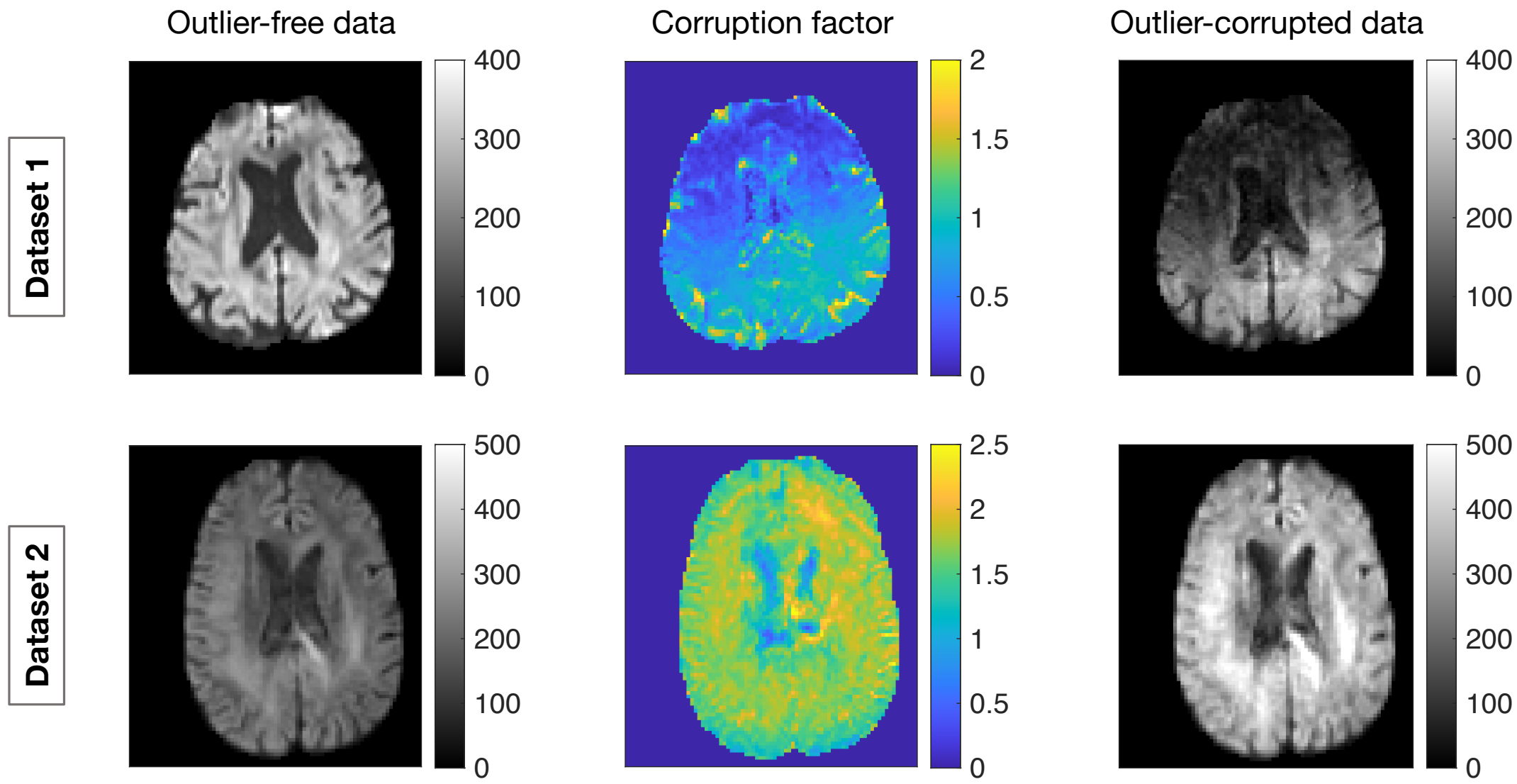

Figure S1. Approach for realistic synthetic data generation for Datasets 1 (top row) and 2 (bottom row). The left column shows the outlier-free data for the selected slices. The middle column displays the voxel-wise corruption factor realistically derived from FSL *eddy* preprocessing. The right column shows the outlier-corrupted data prior to noise injection, obtained by element-wise multiplication of the outlier-free synthetic dataset with the corruption factor.

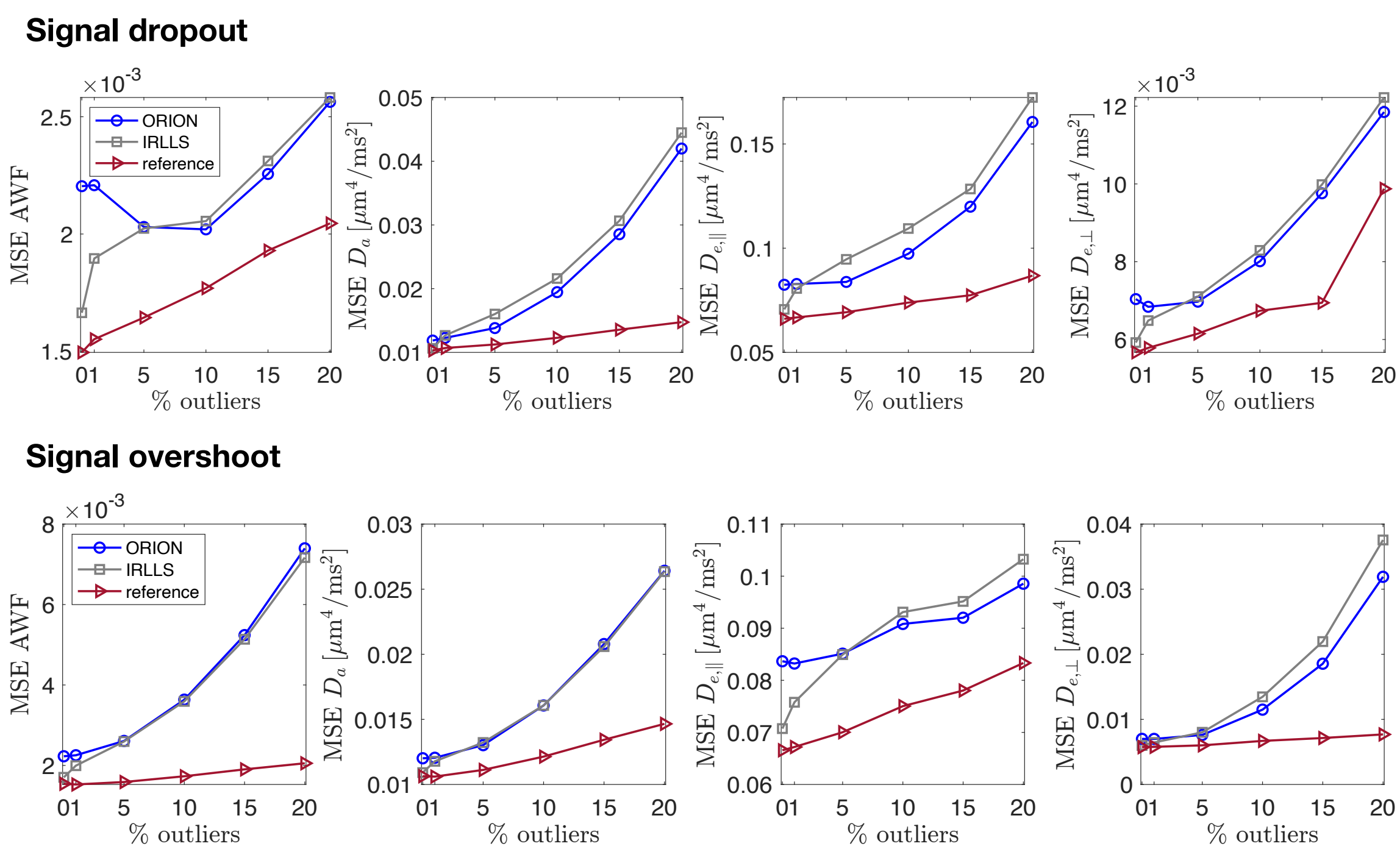

Figure S2. Mean squared error (MSE) of AWF, D_a, $D_{e,\parallel}$ and $D_{e,\perp}$ as a function of the percentage of outliers (% outliers) for IRLLS, ORION and the "reference" approach. The different methods are compared for both outliers manifesting as signal losses (top row) and outliers manifesting as signal overshoots (bottom row).

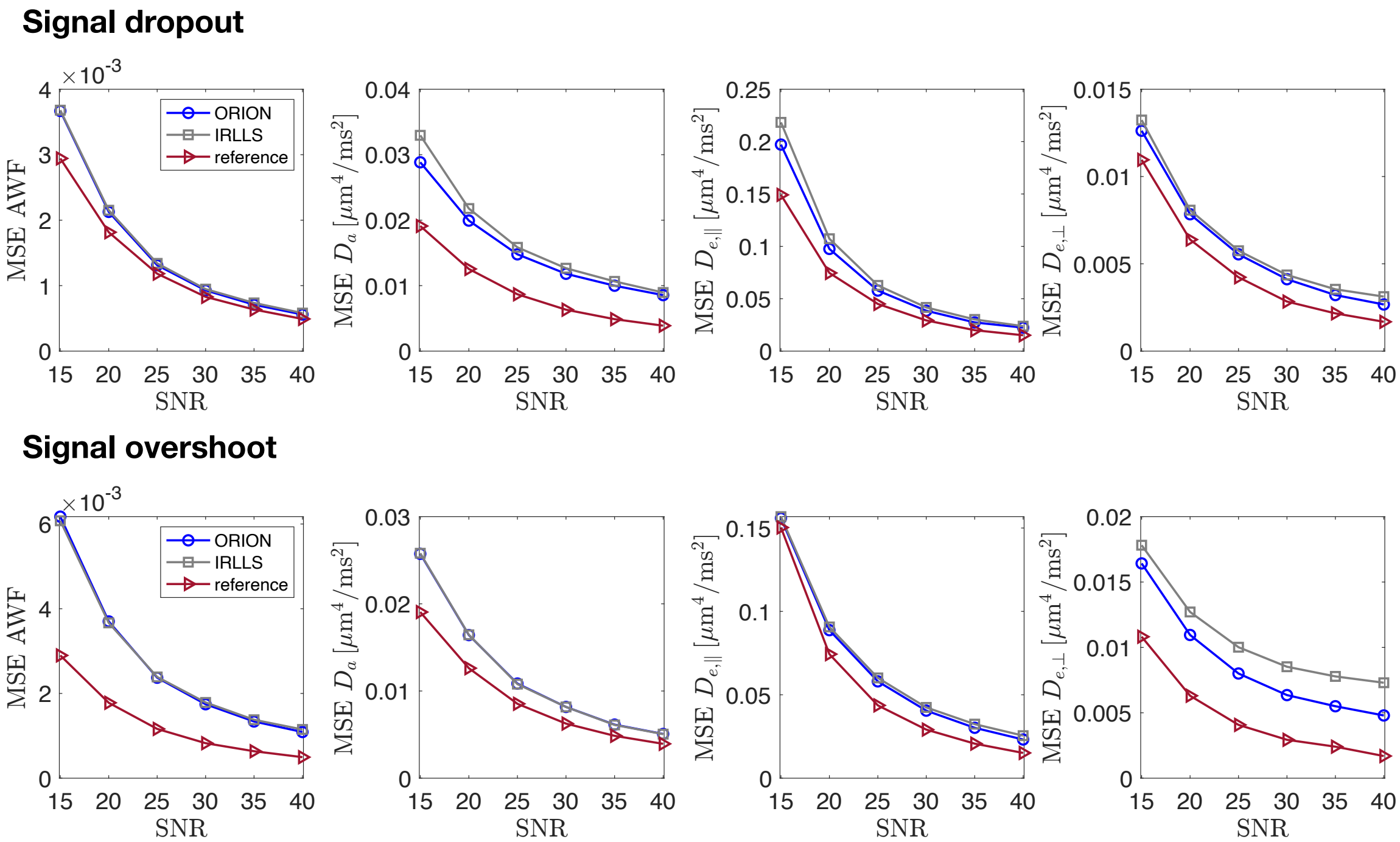

Figure S3. Mean squared error (MSE) of AWF, D_a, $D_{e,\|}$ and $D_{e,\perp}$ as a function of the SNR for IRLLS, ORION and the "reference" approach. The different methods are compared for both outliers manifesting as signal losses (top row) and outliers manifesting as signal overshoots (bottom row).

Dataset	Metric	WLLS	IRLLS	ORION
Dataset 1	$\text{med}_i(\lvert\widehat{\text{AWF}}_i - \text{AWF}_{\text{GT},i}\rvert)$	0.0201	0.0159^a	$0.0155^{a,b}$
	$\text{med}_i(\lvert\widehat{\text{D}}_{a_i} - \text{D}_{a_{\text{GT},i}}\rvert)$ [μm^2/ms]	0.0867	0.0705^a	0.0696^a
	$\text{med}_i(\lvert\widehat{\text{D}}_{e,\|_i} - \text{D}_{e,\|_{\text{GT},i}}\rvert)$ [μm^2/ms]	0.1184	0.0628^a	$0.0616^{a,b}$
	$\text{med}_i(\lvert\widehat{\text{D}}_{e,\perp_i} - \text{D}_{e,\perp_{\text{GT},i}}\rvert)$ [μm^2/ms]	0.0485	0.0381^a	$0.0376^{a,b}$
Dataset 2	$\text{med}_i(\lvert\widehat{\text{AWF}}_i - \text{AWF}_{\text{GT},i}\rvert)$	0.0197	0.0167^a	0.0167^a
	$\text{med}_i(\lvert\widehat{\text{D}}_{a_i} - \text{D}_{a_{\text{GT},i}}\rvert)$ [μm^2/ms]	0.0861	0.0816^a	0.0817^a
	$\text{med}_i(\lvert\widehat{\text{D}}_{e,\|_i} - \text{D}_{e,\|_{\text{GT},i}}\rvert)$ [μm^2/ms]	0.1023	0.0891^a	0.0897^a
	$\text{med}_i(\lvert\widehat{\text{D}}_{e,\perp_i} - \text{D}_{e,\perp_{\text{GT},i}}\rvert)$ [μm^2/ms]	0.0897	0.0562^a	0.0561^a

[a] Significant difference compared to WLLS ($p < 0.0042$)

[b] Significant difference compared to IRLLS ($p < 0.0042$)

Table S1. Median absolute error of AWF, D_a, $D_{e,\|}$ and $D_{e,\perp}$ within a WM mask for Datasets 1 and 2. The median is computed across N_S voxels from the selected axial slices ($i = 1,\ldots,N_S$), where $N_S = 1896$ for Dataset 1 and $N_S = 2149$ for Dataset 2. For each dataset, statistical significance was assessed using a two-sided Wilcoxon signed-rank test with Bonferroni correction for multiple comparisons ($\alpha = 0.05/12$).

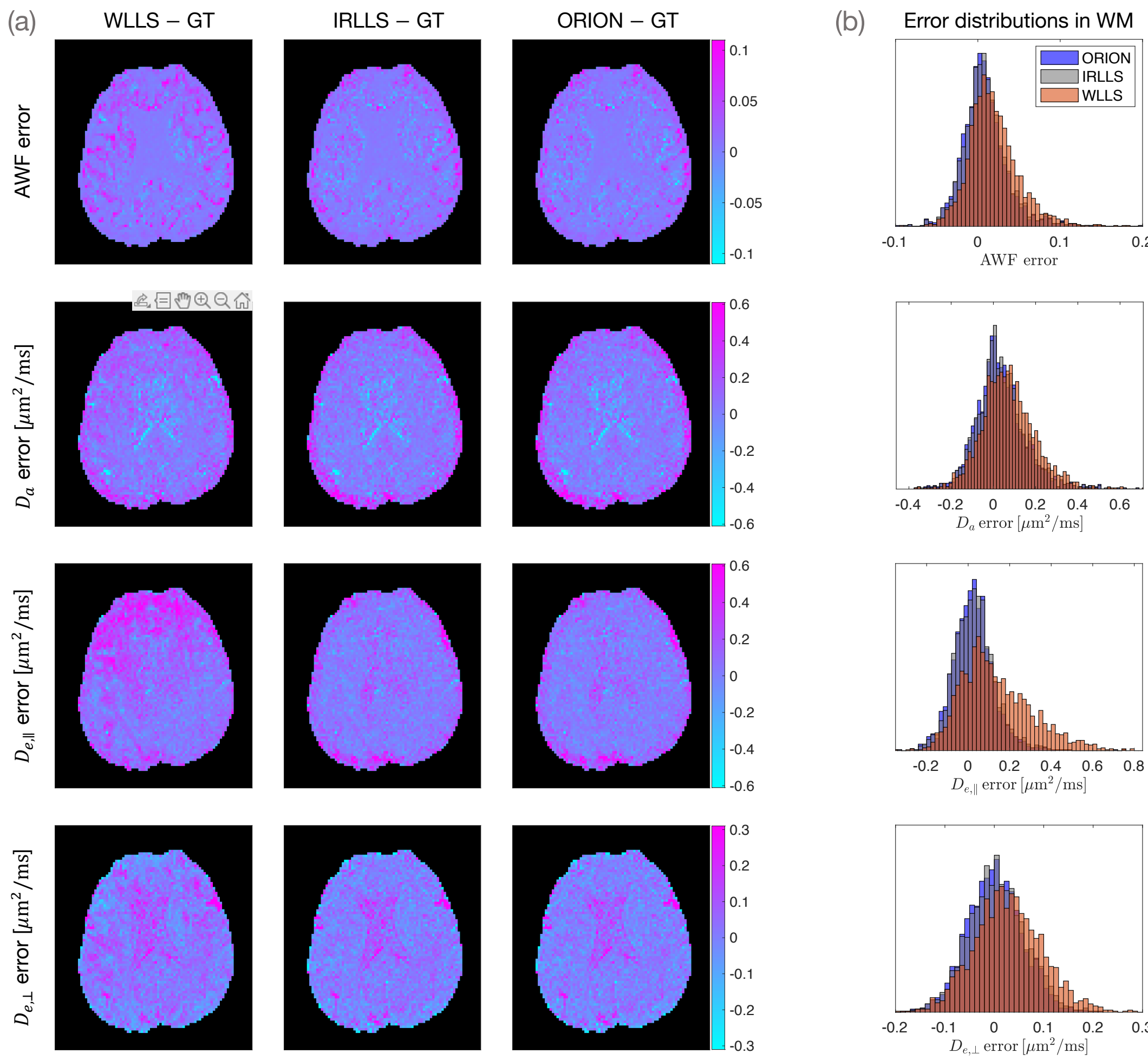

Figure S4. Performance comparison of white matter tract integrity (WMTI) metrics on Dataset 1. **(a)** Voxel-wise error maps for AWF, D_a, $D_{e,\|}$, and $D_{e,\perp}$ across an outlier-corrupted axial slice of the synthetic dataset. The maps are visually compared for the three parameter estimation approaches under assessment: WLLS (left), IRLLS (middle), and ORION (right). **(b)** Error distributions for the same metrics and the same slice restricted to white matter (WM). The distributions are reported for the conventional WLLS approach and for the two outlier-robust approaches, IRLLS and ORION.

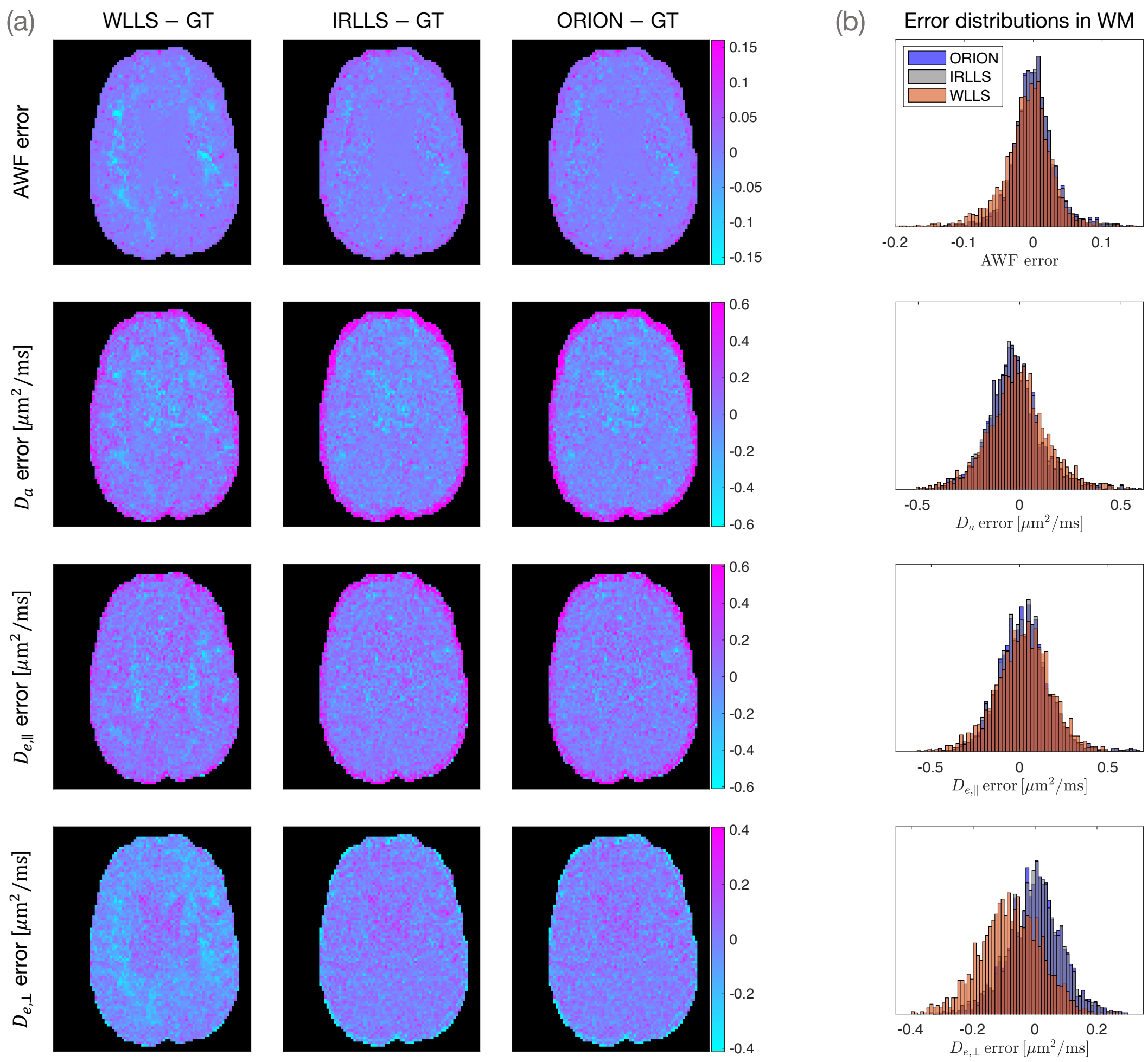

Figure S5. Performance comparison of white matter tract integrity (WMTI) metrics on Dataset 2. **(a)** Voxel-wise error maps for AWF, D_a, $D_{e,\parallel}$, and $D_{e,\perp}$ across an outlier-corrupted axial slice of the synthetic dataset. The maps are visually compared for the three parameter estimation approaches under assessment: WLLS (left), IRLLS (middle), and ORION (right). **(b)** Error distributions for the same metrics and the same slice restricted to white matter (WM). The distributions are reported for the conventional WLLS approach and for the two outlier-robust approaches, IRLLS and ORION.

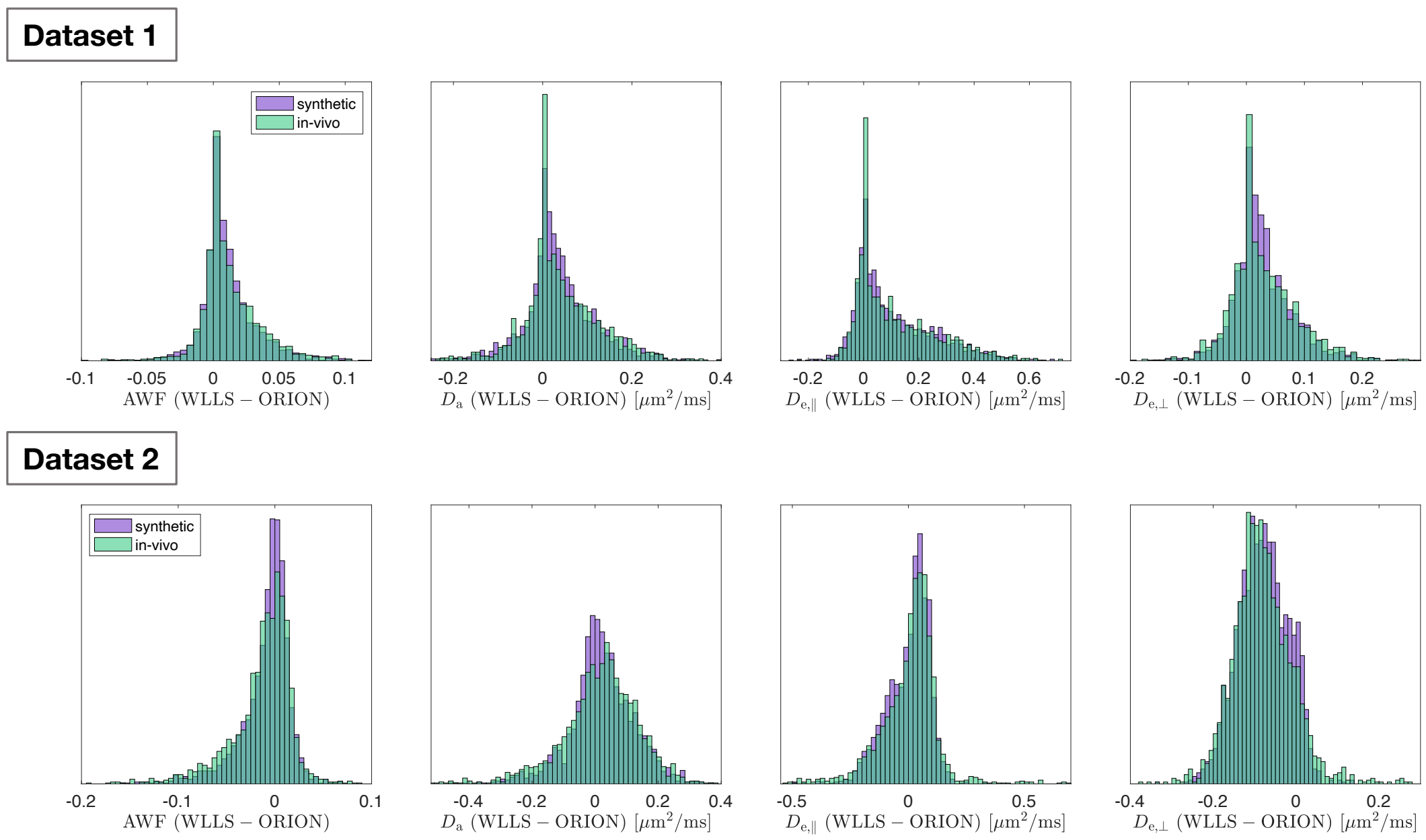

Figure S6. Distributions of the difference between WLLS and ORION estimates of AWF, D_a, $D_{e,\parallel}$, and $D_{e,\perp}$ within a WM mask. Results are shown for an outlier-corrupted axial slice of both synthetic and in vivo datasets: Dataset 1 (top row) and Dataset 2 (bottom row).

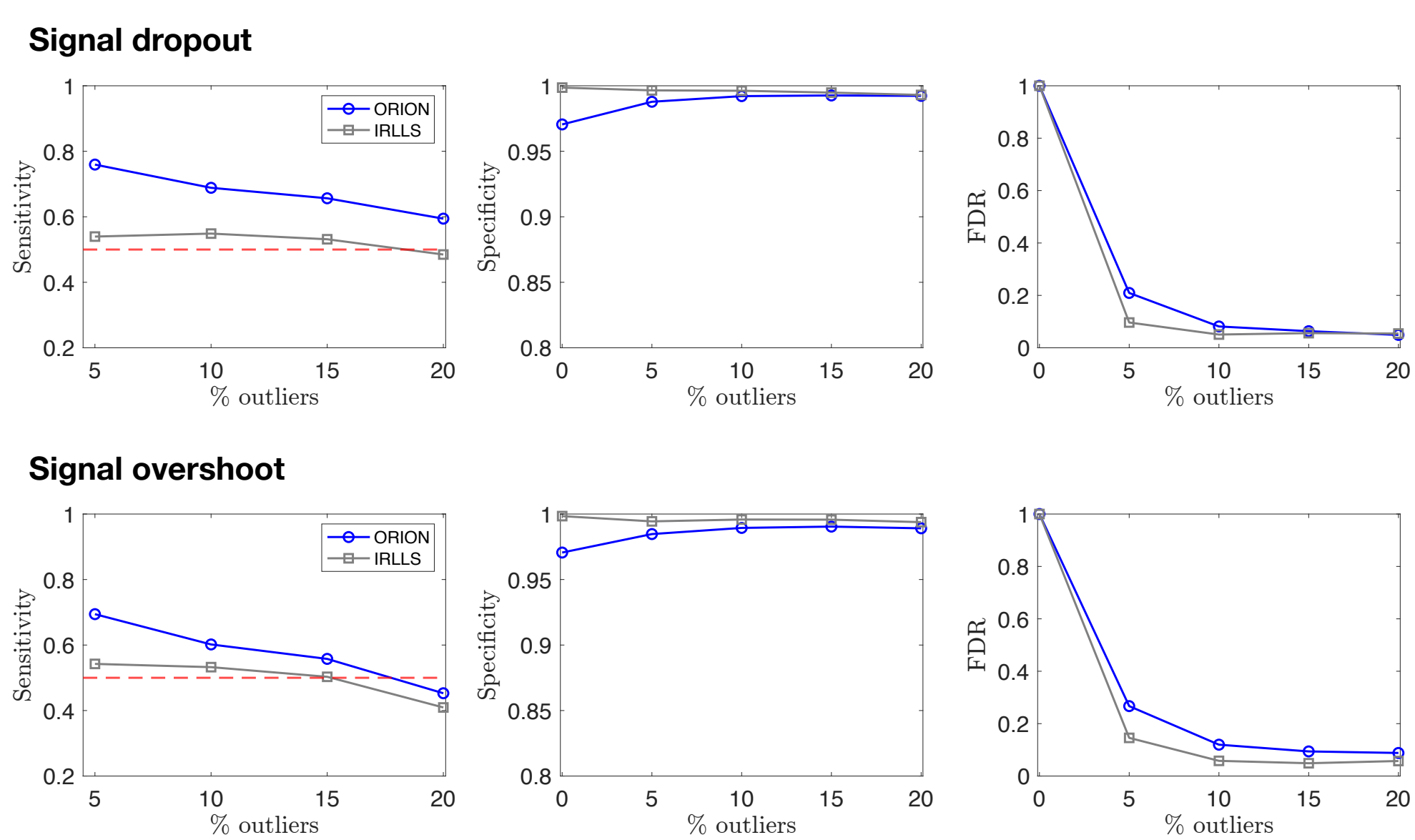

Figure S7. Sensitivity, specificity, and false discovery rate (FDR) of the outlier detection step as a function of the percentage of outliers (% outliers) for DTI signal representation. IRLLS and ORION are compared for both outliers manifesting as signal losses (top row) and outliers manifesting as signal overshoots (bottom row). The red dashed line corresponds to a 50% sensitivity.

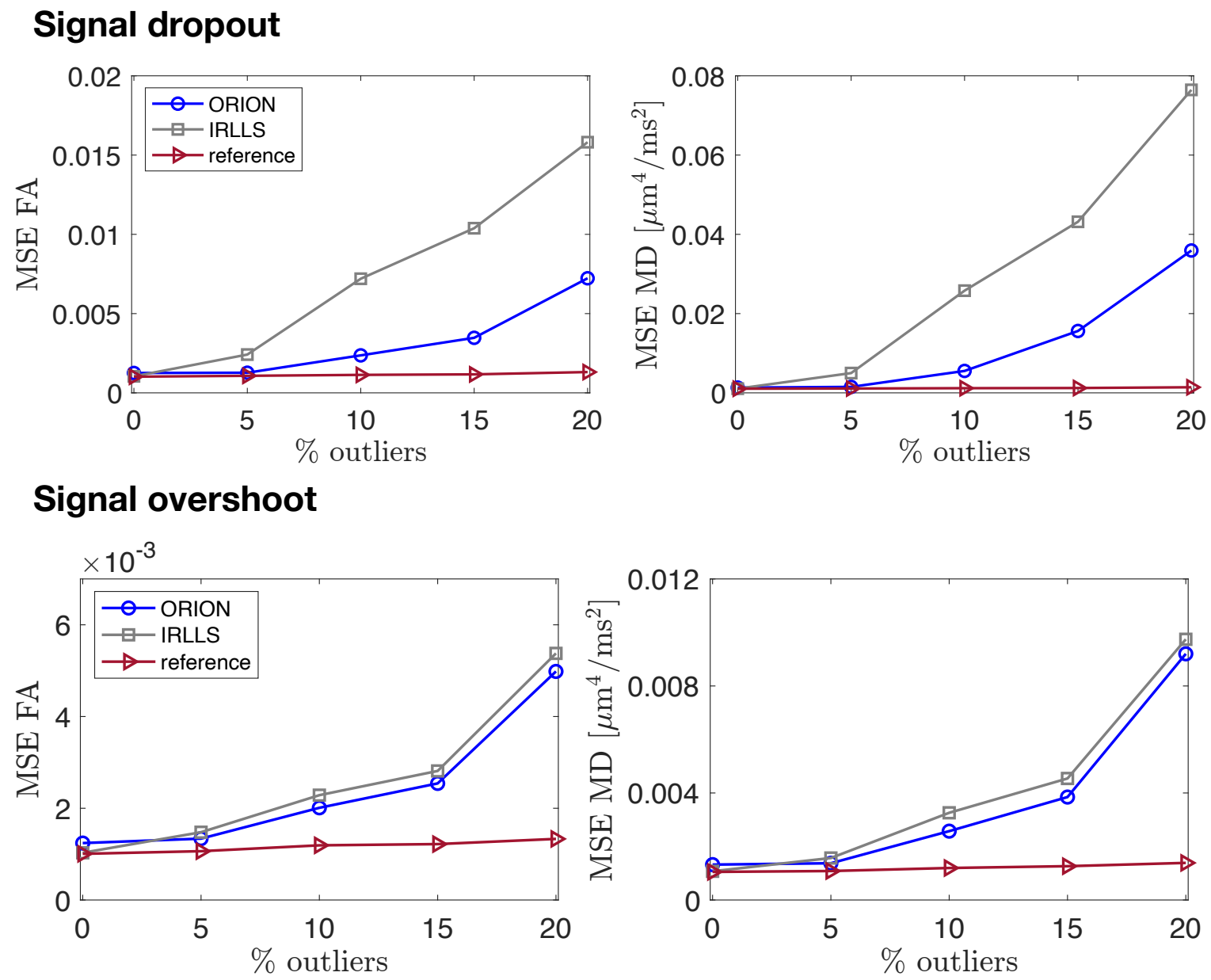

Figure S8. Mean squared error (MSE) of FA and MD as a function of the percentage of outliers (% outliers) for DTI signal representation. IRLLS, ORION and the "reference" approach are compared for both outliers manifesting as signal losses (top row) and outliers manifesting as signal overshoots (bottom row).

Supplementary References

1. Garyfallidis, E. *et al.* Dipy, a library for the analysis of diffusion MRI data. *Front. Neuroinformatics* **8**, 8, DOI: https://doi.org/10.3389/fninf.2014.00008 (2014).
2. Collier, Q., Veraart, J., Jeurissen, B., den Dekker, A. J. & Sijbers, J. Iterative reweighted linear least squares for accurate, fast, and robust estimation of diffusion magnetic resonance parameters. *Magn. Reson. Medicine* **73**, 2174–2184, DOI: https://doi.org/10.1002/mrm.25351 (2015).
3. Tax, C. M., Otte, W. M., Viergever, M. A., Dijkhuizen, R. M. & Leemans, A. REKINDLE: Robust Extraction of Kurtosis INDices with Linear Estimation. *Magn. Reson. Medicine* **73**, 794–808, DOI: https://doi.org/10.1002/mrm.25165 (2015).
